# The performance of mid-upper arm circumference for identifying children and adolescents with overweight and obesity: a systematic review and meta-analysis

**DOI:** 10.1017/S1368980022000143

**Published:** 2022-03

**Authors:** Binyam Girma Sisay, Hamid Yimam Hassen, Beshada Rago Jima, Evan Atlantis, Seifu Hagos Gebreyesus

**Affiliations:** 1 Department of Nutrition and Dietetics, School of Public Health, Addis Ababa University, Addis Ababa, Ethiopia; 2 Department of Primary and Interdisciplinary Care, Faculty of Medicine and Health Sciences, University of Antwerp, Antwerp, Belgium; 3 School of Health Sciences, Western Sydney University, Penrith, NSW, Australia; 4 Discipline of Medicine, Nepean Clinical School, Faculty of Medicine and Health, The University of Sydney, Nepean, NSW, Australia

**Keywords:** Mid-upper arm circumference, ROC curve, Overweight, Obesity, Children and adolescents

## Abstract

**Objective:**

This study aimed to synthesise the existing evidence on the performance of mid-upper arm circumference (MUAC) to identify children and adolescents with overweight and obesity.

**Design:**

Systematic review and meta-analysis.

**Setting:**

We searched PubMed, EMBASE, SCOPUS, Cochrane Library, Web of Science, CINAHL and Google scholar databases from their inception to December 10, 2021, for relevant studies. There were no restrictions regarding the language of publication. Studies reporting measures for the diagnostic performance of MUAC compared with a reference standard for diagnosing overweight and obesity in children and adolescents aged 2–19 years were included.

**Participants:**

A total of 54 381 children and adolescents from twenty-one studies were reviewed; ten studies contributed to meta-analyses.

**Results:**

In boys, MUAC showed a pooled AUC of 0·92 (95 % CI 0·89, 0·94), sensitivity of 84·4 (95 % CI 84·6, 90·8) and a specificity of 86·0 (95 % CI 79·2, 90·8), when compared against BMI z-score, defined overweight and obesity. As for girls, MUAC showed a pooled AUC of 0·93 (95 % CI 0·90, 0·95), sensitivity of 86·4 (95 % CI 79·8, 91·0), specificity of 86·6 (95 % CI 82·2, 90·1) when compared against overweight and obesity defined using BMI z-scores.

**Conclusion:**

In comparison with BMI, MUAC has an excellent performance to identify overweight and obesity in children and adolescents. However, no sufficient evidence on the performance of MUAC compared with gold standard measures of adiposity. Future research should compare performance of MUAC to the ‘golden standard’ measure of excess adiposity.

Overweight and obesity in childhood and adolescence is one of the greatest public health problems facing most countries in the world, which has increased dramatically in recent decades^(1,2)^. The global prevalence of obesity in children and adolescents was 5·6 % in girls and 7·8 % in boys, respectively, in 2016^(2)^. Childhood obesity can persist into adulthood, leading to an increased risk of chronic non-communicable diseases and premature mortality^(3–6)^. Timely recognition and early diagnosis of overweight and obesity in young people are essential to mitigate the short- and long-term health risks associated with excess body weight, especially fat mass^(7,8)^. Health care professionals need to use an accurate and efficient screening tool to diagnose overweight and obesity for early intervention^(9)^.

According to the WHO, obesity is defined as an abnormal or excessive fat accumulation that may impair health^(10)^. Total body fat can be accurately measured using several methods such as hydrostatic weighing, air displacement plethysmography, deuterium oxide dilution or dual-energy X-ray absorptiometry, which estimates total body fat by measuring total body water and based on the absorption patterns of X-rays^(11,12)^. However, these methods are laboratory-based, expensive, time-consuming and not feasible for routine use^(13)^.

Alternatively, there are a range of anthropometric measurements that are simple and inexpensive options for overweight and obesity screening in children and adolescents^(14)^. However, the results of anthropometric measurements need to be interpreted using reference standards to define overweight and obesity in young people. While the BMI z-score is a widely utilised method to identify those with overweight and obesity in epidemiological studies but has limited applicability in routine clinical practice^(2,15)^. Simple and inexpensive alternatives to BMI z-scoring would be helpful to promote screening and early identification, especially in low- and middle-income countries with limited health care resources^(16)^.

The mid-upper arm circumference (MUAC) has been proposed as one such alternative to screen for overweight and obesity in children and adolescents^(17–20)^. It is a simple measure commonly used to screen for undernutrition in infants and children aged 6–59 months^(21)^ as well as thinness and severe thinness in adolescents^(22,23)^.The existing evidence of the usefulness of the MUAC against the BMI Z-score among children and adolescents is limited and unclear^(17–20,24–26)^. Therefore, this systematic review and meta-analysis aim to summarise the currently available evidence on the performance of MUAC to identify children and adolescents with overweight and obesity.

## Methods

This systematic review and meta-analysis were conducted per the Preferred Reporting Items for Systematic Reviews and Meta-Analyses for Diagnostic Test (PRISMA-DTA) statement (see online supplementary material, Supplemental Table S1)^(27)^. The protocol of this systematic review and meta-analysis was registered on PROSPERO (Registration number CRD42020183148) and published^(28)^; minor deviations from the original protocol have also been explained (see online supplementary material, Supplemental Table S2).

### Review question

This systematic review aims to generate an evidence summary to answer the following review question: what is the diagnostic performance of the MUAC assessment for diagnosing overweight and obesity in children and adolescents.

### Search strategy and study selection

A systematic search was performed in PubMed, EMBASE, SCOPUS, Cochrane Library, Web of Science and CINAHL database of references for peer-reviewed articles. To retrieve grey literature, systematic search was performed using Google Scholar. The databases were systematically searched from their inception to December 10, 2021. A detailed search strategy is provided in the supplementary material (see online supplementary material, Supplemental Table S3).

All articles identified through the systematic search databases were imported into EndNote as a single library. Duplicate articles from the searches were verified and removed. The remaining articles were imported into rayyan.QCRI.org^(29)^, a web-based tool that facilitates screening and collaboration among researchers.

Two independent reviewers (BGS and BRJ) conducted the title and abstract screening and included articles for the full-text review. Disagreement between the two reviewers was resolved by inviting the third reviewer (HYH) to make the final decision. The following inclusion criteria were used:

1. Population: children or adolescents aged 2–19 years.
2. Index test: studies that assessed the diagnostic performance of MUAC as an index test to identify children and adolescents with overweight/obesity.
3. Comparator: compared to reference standards such as BMI z-score, weight to height, waist circumference, skinfold thickness, dual-energy X-ray absorptiometry, air-displacement plethysmography, bioelectrical impedance and hydro densitometry.
4. Outcome: overweight and obesity.
5. Study design: observational studies including cross-sectional, cohort and case-control were included.
6. Language: studies published in any language were included.
7. Year of publication: no restriction was made based on the year of publication.

Studies that fulfill any of the following criteria were excluded:

1. Articles available only in abstract form, letters, reviews, commentaries, editorials, case series.
2. Duplicate publication of the same study

### Data extraction

Two independent reviewers (BGS and BRJ) have extracted the following information from included studies, using pilot-tested data collection form: first author’s name, year of publication, country or region, funding source, study design, total sample size, number of males and females, response rate, age of study participants, MUAC cut-off values, reference standard, diagnostic criteria of overweight and obesity (reference standard), sensitivity, specificity, AUC, positive and negative likelihood ratio, prevalence of overweight and obesity, positive and negative predictive values. The extracted data by independent reviewers were compared and any discrepancy was resolved by consensus. When relevant information was missing from the article, we contacted the primary authors twice via e-mail.

### Risk of bias and certainty of evidence assessment

Two independent reviewers (BGS and BRJ) assessed the risk of bias and applicability of the included studies using Quality Assessment of Diagnostic Accuracy Studies (QUADAS-2)^(30)^. We have also assessed the certainty of evidence for relevant outcomes using the Grading of Recommendations Assessment, Development and Evaluation (GRADE) approach for diagnostic tests^(31)^. Discrepancies among reviewers on individual items were resolved by discussion and consensus.

### Statistical analysis

The extracted data were exported to STATA/SE Version 16 for further processing and analysis. We have summarised the diagnostic test accuracy by creating a 2 × 2 table for each study. We have performed a graphical descriptive analysis of the included studies. We have reported coupled forest plots (sensitivity and specificity separately, along with the 95 % CI), and we provided a graphical representation of studies in the summary receiver operating characteristic (SROC) curve (sensitivity against 1 – specificity).

Among the included studies, those that report the number of true positive, true negative, false positive, false negative or values that are required to calculate them are included in the meta-analysis.

We have used the hierarchical summary receiver-operating characteristic curve model to produce SROC curves^(32)^. The AUC – SROC curve values were used to describe test accuracy. We evaluated the discriminatory power by the AUC-SROC using values proposed by Swets^(33)^, with ≤ 0·5 considered to have no discriminatory power, > 0·5 and ≤ 0·7 to have low discriminatory power, > 0·7 and ≤ 0·9 to have good discriminatory power and 1 to be a perfect test. In addition, we have estimated the pooled sensitivity, specificity, positive likelihood ratio and negative likelihood ratio to complement the findings of SROC. Subgroup analysis was performed for boys, girls, children (2–9 years), adolescents (10–19 years), with overweight and obesity.

We assessed the heterogeneity of diagnostic test parameters by visual inspection of the paired forest plots and SROC plots. One of the major sources of heterogenicity in diagnostic accuracy systematic review and meta-analysis is the use of different thresholds. We have assessed the presence of a threshold effect using spearman’s correlation coefficient between the logit of sensitivity and the logit of 1 – specificity^(34)^. We have explored potential sources of bias including sex, cut-off point, age group and weight status (overweight, obesity) variables. To further investigate heterogeneity, we performed subgroup analysis for boys, girls, children (2–9 years), adolescents (10–19 years), children and adolescents with overweight, children and adolescents with obesity. To assess possible publication bias, we used Deeks’ funnel plot, with Deeks’ asymmetry test, where *P* < 0·05 was considered as significant asymmetry^(35)^.

## Results

### Selection of studies

**Fig. 1:**
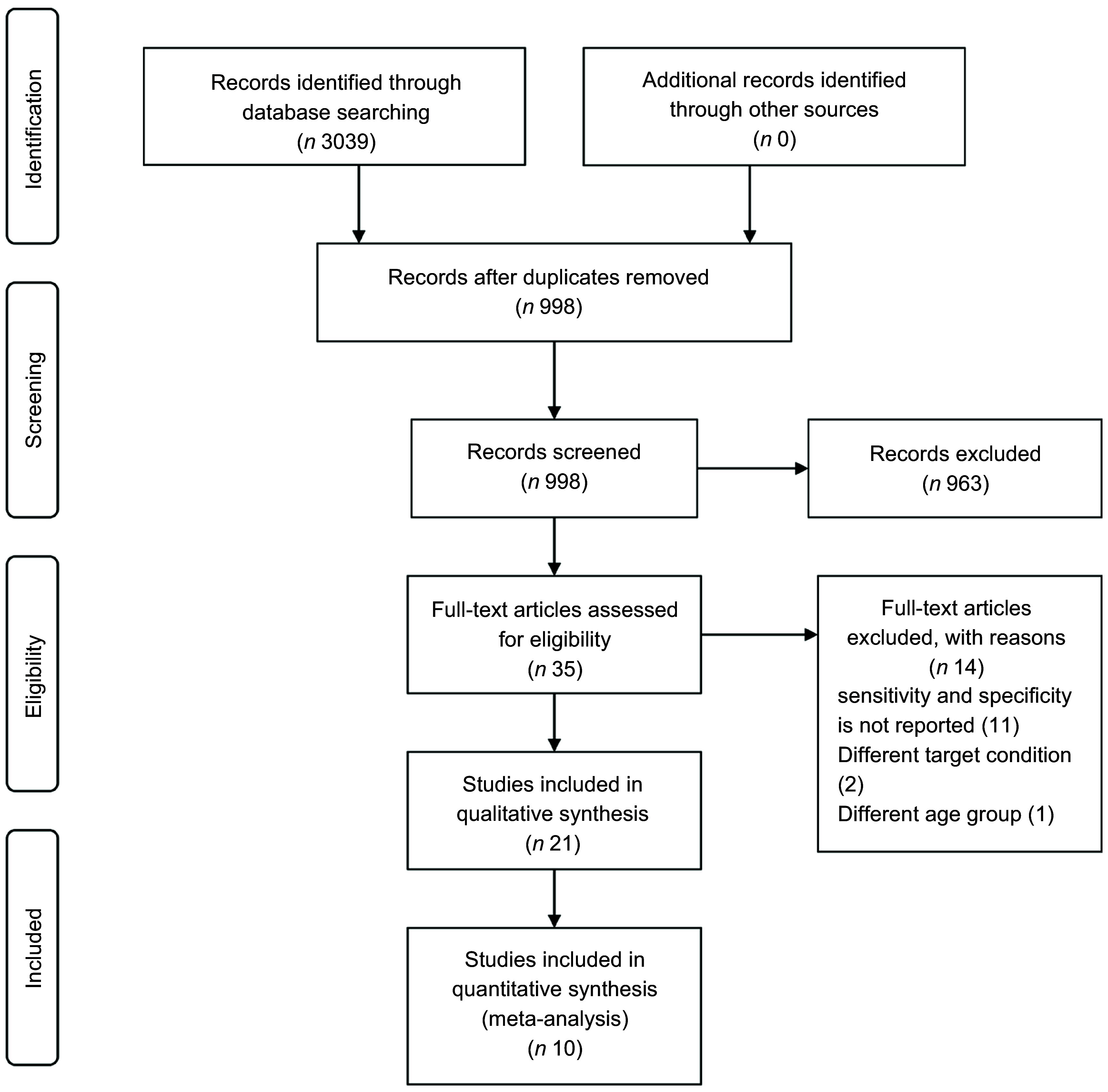
Study selection flow chart

The search strategy results in a total of 3039 references. Of these, 2041 were duplicates, resulting in 998 articles. After screening titles and abstracts, 963 studies were excluded. Thirty-five papers were retained for review after full-text evaluation. Of these, fourteen articles were excluded with reasons; did not report measures of diagnostic performance; different target condition; was conducted on age group outside of the scope of this review. Therefore, twenty-one studies met the inclusion criteria and were included in the systematic review, however, ten studies with sufficient information are included in the meta-analysis. These eleven articles reported neither the required parameters for meta-analyses (true positive, true negative, false negative or false positive) nor other parameters to calculate them (prevalence, positive predictive value or negative predictive value). Figure 1 shows the detailed description of the article screening process.

### Characteristics of studies

Table 1 shows characteristics of studies included in the systematic review. All of the studies were cross-sectional and were published from 2013 to 2021. They were conducted in twenty-one countries including Brazil^(36)^, China^(37)^, Netherlands^(38)^, Ethiopia^(20)^, India^(39–41)^, Indonesia^(42)^, Nigeria^(43,44)^, Pakistan^(25,45)^, Seri Lanka^(24)^, South Africa^(46,47)^, Thailand^(48)^, Turkey^(49,50)^, Trinidad and Tobago^(51)^ and twelve countries^(17)^ (Australia, Brazil, Canada, China, Colombia, Finland, India, Kenya, Portugal, South Africa, the UK and the USA) (*n* 1). The number of participants varied substantially between studies (range from 211 to 31 471), with a pooled population of 54 381 children and adolescents.

**Table 1:** Characteristics of included studies

Study author	Location	Study design	Sample size	Boys/girls	Sex	Age	Reference standard	Outcome threshold	Cut-off	Sensitivity	Specificity
Lu *et al.* ^(26)^ (2013)	China	Cross-sectional	2847	1475/1372	Boys and girls	7–12	BMI	≥ 85th percentiles	18·9–23·4	83–95 %	82–96 %
Rerksuppaphol & Rerksuppaphol^(57)^ (2017)	Thailand	Cross-sectional	3618		Boys and girls	7–12	BMI	BMI Z score > 2 + sd	19·8–25·5	82–96 %	89·2–97 %
					Boys and girls	8		BMI Z score > 1 + sd	18·0–23·2	93 %	94 %
Asif *et al.* ^(25)^ (2018)	Pakistan	Cross-sectional	7921	4021/3900	Boys and girls	5–14	BMI	BMI Z score > +1sd	16·38–22·73	60–90 %	59–90 %
Craig *et al.* ^(18)^ (2014)	South Africa	Cross-sectional	978		Boys and girls	5–14	BMI	BMI Z score > +1sd		64–97 %	76–93 %
					Boys and girls	5–14	BIA	> 85th percentile		26–100 %	73–95 %
Chaput *et al.* ^(17)^ (2016)	12 countries	Cross-sectional	7337	3408/3929	Boys and girls	9–11	BMI	BMI Z score > +2sd	24·6–25·2	94–95 %	90–92 %
Jaiswal *et al.* ^(39)^ (2017)	India	Cross-sectional	875	436/439	Boys and girls	5–14	BMI		18·8–23·3	91–100 %	95·1–100 %
Talma *et al.* ^(19)^ (2018)	Dutch	Cross-sectional	6167		Boys and girls	2–18	BMI	≥ 85th percentiles	>1·3 SDS	52–95 %	71–94 %
Mazıcıoğlu *et al.* ^(58)^ (2010)	Turkey	Cross-sectional	5358	2621/2737	Boys and girls	6–17	BMI	≥ 85th percentiles	18·1–24·9	50–95 %	76–98 %
					Boys and girls	6–17	WC	≥ 90th percentile	17·9–25·7	53–100 %	63–92 %
Ayu *et al.* ^(42)^ (2017)	Indonesia	Cross-sectional	2258		Boys and girls	6–7	BMI	≥ 85th percentile	18·5	88–89 %	78 %
					Boys and girls	6–7		≥ 95th percentile	19·5	85–93 %	86–87 %
					Boys and girls	6–7		≥ 85–< 95 percentile	18·5	73–83 %	78 %
Shinsugi *et al.* ^(24)^ (2020)	Serilanka	Cross-sectional	528		Boys and girls	5–10		BMI Z score > +1 sd	19·05–21·8	95–100 %	85–96 %
Dumith *et al.* ^(36)^ (2018)	Brazil	Cross-sectional	1075	512/563	Boys and girls	13–19	BMI	BMI Z score > +1sd		58–84 %	57–89 %
Otitoola *et al.* ^(59)^ (2020)	South Africa	Cross-sectional	211	102/109	Boys and girls	6–19		BMI Z score > +1sd			
Orimadegun ^(43)^ (2019)	Nigeria	Cross-sectional	920	403/517	Boys and girls	5–18		BMI Z score > +2sd		58–94 %	20–88 %
Asif *et al.* ^(45)^ (2018)	Pakistan	Cross-sectional	4962		Boys and girls	12–18	BMI	≥ 85th percentile	19·43–24·76	47–84 %	62–85 %
de Almeida *et al.* ^(60)^ (2003)	Brazil	Cross-sectional	1090		Boys and girls		BMI	0·7 (z score)	0·6–0·7 (z score)	77–79 %	78 %
Oriaifo *et al.* ^(44)^ (2019)	Nigeria	Cross-sectional	1067	538/529	Boys and girls	6–18	BIA	≥ 85th percentile	18·75–30·0	60–100 %	53–100 %
Sisay *et al.* ^(20)^ (2020)	Ethiopia	Cross-sectional	851	456/395	Boys and girls	15–19	BMI	BMI Z score > +1 sd	27·75–27·95	90–94 %	89–91 %
Khiamniungan & Mondal ^(40)^ (2019)	India	Cross-sectional	960	400	Boys and girls	6–16	BMI	BMI Z score > +1 sd	16·5–21·6	50–95 %	65–80 %
Ozturk *et al.* ^(52)^ (2015)	Turkey	Cross-sectional	5358	968	Boys	6–17	WC	WC ≥ 90th percentile	9·5		
Ramcharitar-Bourne *et al.* ^(51)^ (2021)	Trinidad and Tobago	Cross-sectional	595	301/295	Boys and girls		BIA	≥ 85th percentile	MUAC z-score	43–92 %	95–98 %
Nitika ^(41)^ (2021)	India	Cross-sectional	31 471	16 158/15 313	10–19	BMI	BMI	BMI Z score > +1sd	21·2–29·8	74·5–90 %	74·5–90 %

BIA, bioelectrical impedance analysis; WC, waist circumference.

Studies used different reference methods: three studies used bioelectrical impendency^(18,44)^; two used waist circumferences^(49,52)^; the rest used BMI. Most studies used an 85th percentile (Z score > 1 + sd) cut-off of BMI growth curves for overweight and a 95th percentile cut-off (Z score > 2 + sd) of BMI curve for obesity. Both studies that use bioelectrical impendency classify participants with 85th percentile as overweight^(18,44)^. The cut-off points of MUAC values for defining overweight and obesity varied substantially between studies (see online supplementary material, Supplemental Table S4).

### Meta-analysis

**Fig. 2:**
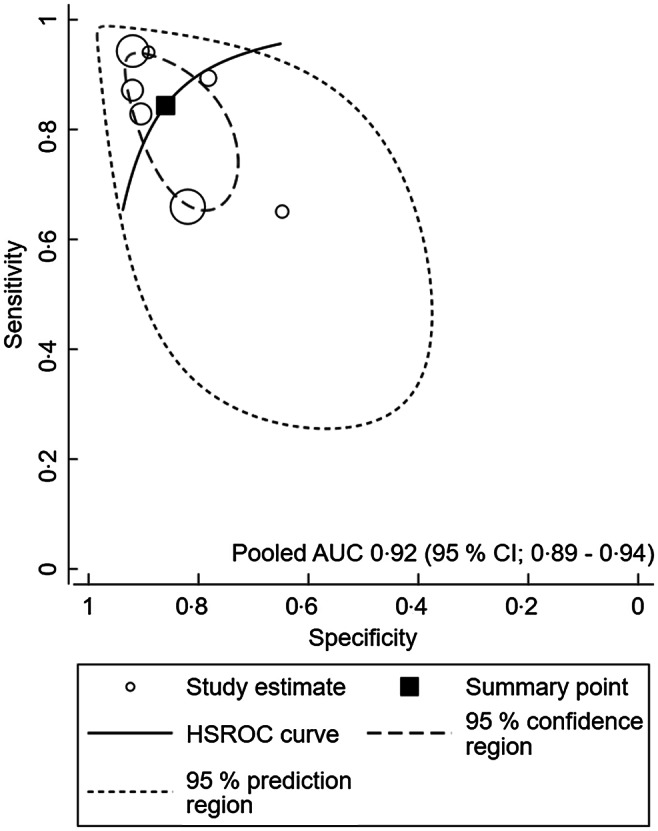
Summary receiver operating characteristic (SROC) curve for boys

**Fig. 3:**
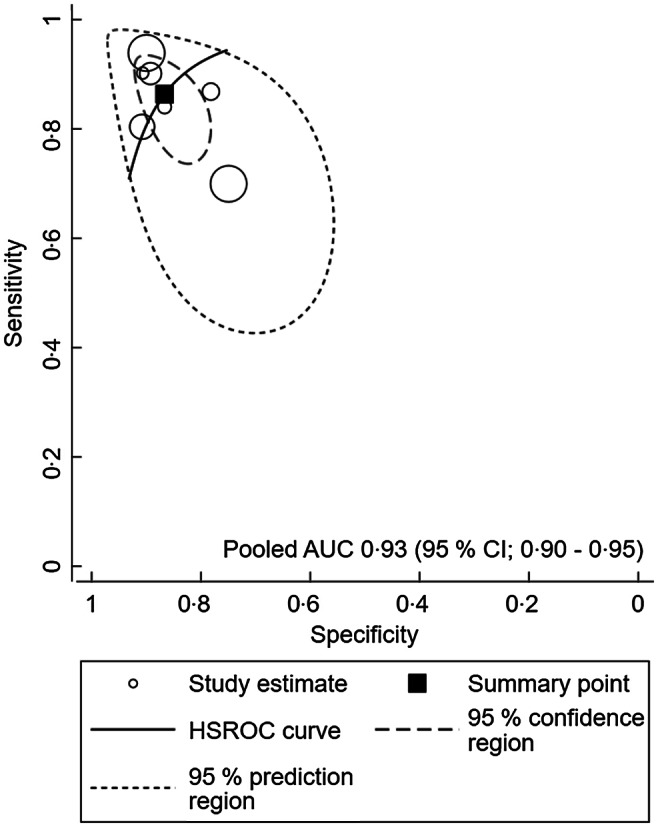
Summary receiver operating characteristic (SROC) curve for girls

A meta-analysis was conducted to assess the performance of MUAC for identifying overweight and obesity in children and adolescents. The meta-analysis showed that a pooled AUC of 0·92 (95 % CI 0·89, 0·94), sensitivity of 85·2 (95 % CI 77·3, 90·6) and a pooled specificity of 85·6 (95 % CI 81·0, 98·2) for boys and girls, respectively. In boys, MUAC showed a pooled AUC of 0·92 (0·89, 0·94), sensitivity of 84·4 (95 % CI 84·6, 90·8) and a pooled specificity of 86·0 (95 % CI 79·2, 90·8) (Fig. 2 and Table 2). As for girls, MUAC showed a pooled AUC of 0·93 (95 % CI 0·90, 0·95), sensitivity of 86·4 (95 % CI 79·8, 91·0) and pooled specificity of 86·6 (95 % CI 82·2, 90·1) (Fig. 3 and Table 2).

**Table 2:** Diagnostic performance of mid-upper arm circumference

Sex	Pooled AUC	95 % CI	Pooled sensitivity	95 % CI	Pooled specificity	95 % CI	Positive likelihood ratio	95 % CI	Negative likelihood ratio	95 % CI
Boys	0·92	0·89, 0·94	84·4	84·6, 90·8	86·0	79·0, 90·8	6·0	3·7, 9·7	0·12	0·10, 0·31
Girls	0·93	0·90, 0·95	86·4	79·8, .91·0	86·6	82·2, 90·1	6·5	4·6, 9·1	0·16	0·11, 0·24
Boys and girls	0·92	0·89, 0·94	85·2	77·3, 90·6	85·6	81·0, 89·2	5·9	4·0, 8·9	0·17	0·11, 0·28
2–9	0·88	0·85, 0·90	83·6	67·9, 92·1	78·8	66·1, 87·7	3·9	2·2, 7·2	0·21	0·01, 0·47
10–19	0·96	0·93, 0·97	91·5	87·1, 94·5	88·9	85·5, 91·6	8·3	6·4, 10·7	0·09	0·06, 0·14
Overweight	0·91	0·88, 0·93	84·1	75·3, 90·1	84·3	79·4, 88·3	5·4	3·8, 7·5	0·18	0·11, 0·31
Obesity	0·95	0·92, 0·96	89·4	80·2, 94·6	88·9	83·4, 92·8	8·1	5·0, 16·9	0·12	0·06, 0·24

We have further explored the diagnostic performance of the MUAC by weight status. The highest discriminatory ability was observed among children and adolescents with obesity, resulting in a pooled AUC of 0·95 (95 % CI 0·92, 0·96), sensitivity of 89·4 (95 % CI 80·2, 94·6) and a pooled specificity of 88·9 (95 % CI 83·4, 92·8).

Moreover, we have analysed the diagnostic performance of MUAC by the age of participants 2–9 years and 10–19 years. The MUAC showed higher performance in adolescents between the age of 10–19 compared to those aged 2–9 years with an AUC of 0·96 (0·93, 0·97) *v*. 0·88 (95 % CI 0·85, 0·90).

### Risk of bias and publication bias

The full results of the risk of bias and applicability were assessed using QUADAS-2 is shown in supplementary material (see online supplementary material, Supplemental Table S5). The study design and procedure were homogeneous and almost all met all QUADAS-2 domains. One study was classified as ‘high risk’ in the question of applicability domain (Index test) because the reference population they use to identify children with overweight and obesity was inappropriate. All included studies had a ‘high risk’ of bias domain (reference standard) because they used BMI as their reference standard, which is not a golden standard to measure excess adiposity. Even though BMI is highly correlated with excess adiposity, it misclassifies a significant number of children and adolescents^(9)^. Except for one^(20)^, none of the included studies reported the time interval between performing the index test and reference standard. However, it is unlikely that any time delay between conducting the index test and the reference standard would introduce bias. The description of the index tests and reference standards was adequately reported. Evidence of publication bias was not observed by Deek’s test (*P* = 0·71) (see online supplementary material, Supplemental Fig. S1).

### Investigation of heterogenicity

**Fig. 4:**
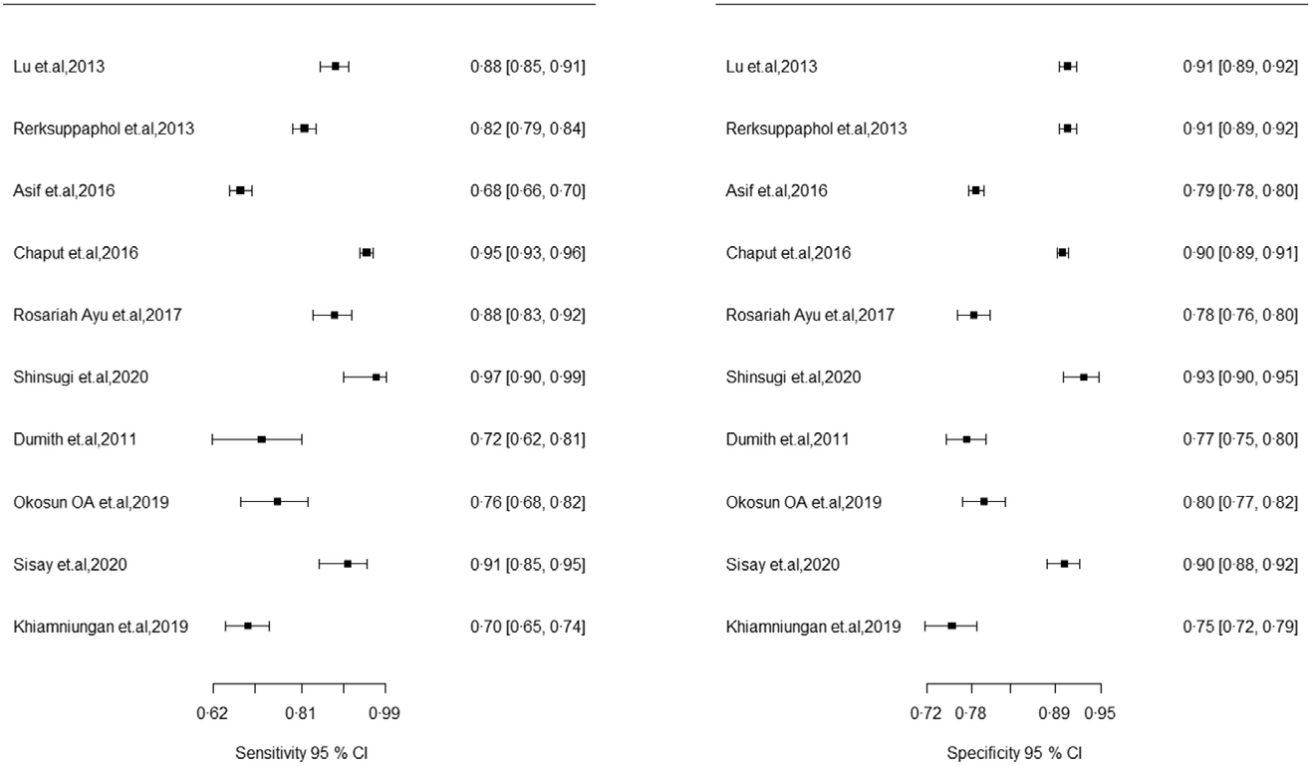
Paired forest plot of sensitivity and specificity with 95 % CI

Visual inspection of the paired forest plot and SROC curve revealed substantial heterogeneity. (Fig. 4) A Spearman’s rank correlation test showed the presence of a threshold effect *r* = 0·63. We have examined the influence of covariate sex, age group and weight status. We have observed that MUAC cut-off (*P*-value = 0·00), age group (*P*-value = 0·00) and weight status (*P*-value = 0·05) significantly contribute to the heterogenicity of the study. However, sex of participants did not contribute to the heterogenicity (*P*-value = 0·10). To further explore sources of heterogeneity, we performed subgroup analyses by sex, age group (children, adolescents) and weight status (overweight, obese). The results suggest that weight status might contribute to the heterogeneity among studies. Since pooling sensitivity and specificity are more reliable in the absence of a threshold effect, the findings should be primarily judged based on the SROC curve.

### Certainty of evidence assessment

We rated the certainty of evidence of the pooled studies and considered it as moderate for all pooled measures of diagnostic accuracy. The reasons for downgrading the certainty of evidence included the marked heterogenicity observed^(31)^. The result of certainty of evidence assessment is available on supplementary material (see online supplementary material, Supplemental Table S6).

## Discussion

This meta-analysis showed that when assessing the diagnostic performance of MUAC compared with BMI defined overweight and obesity. MUAC has high sensitivity and specificity, correctly identifying about 86·4 % of children and adolescents with BMI defined overweight and obese. In addition, MUAC has a greater discriminatory ability in identifying children and adolescents with BMI-defined obesity than those with BMI-defined overweight. However, no sufficient evidence on the performance of MUAC compared with gold standard measures of adiposity.

The MUAC was initially developed to screen and diagnose under-five children with moderate and severe acute malnutrition^(21)^. Moreover, MUAC has also been used to identify adolescents and women of reprobative age with thinness and severe thinness^(22,23)^. In recent years, MUAC has been explored as an alternative screening tool for children and adolescents with overweight and obesity^(17–20,24–26)^. MUAC has attractive characteristics that make it desirable as a simple screening tool.

To the best of our knowledge, this is the first systematic review and meta-analysis to examine the discriminatory performance of MUAC to identify children and adolescents with overweight and obesity considering age group, sex and weight status. The previous systematic review had included MUAC as one of the uncommonly used measures of overweight and obesity. However, this systematic review has not assessed the discriminatory ability of MUAC in identifying children and adolescents with overweight and obesity^(53)^.

This meta-analysis showed that MUAC has a high discriminatory ability to identify both boys and girls with BMI defined overweight and obesity with a comparable value of sensitivity and specificity. However, concerning weight status, MUAC has superior discriminatory ability among children and adolescents with obesity than those who are BMI defined overweight. Similar findings have been reported by a meta-analysis conducted on the performance of neck circumference^(54)^. This might be due to the fact that sensitivity and specificity of screening tools depend on the spectrum of a condition, in this case, the degree of adiposity; those who are obese are more likely to be easily identified by screening tools than those who are overweight.

An ideal anthropometric measurement to identify adolescents with overweight and obesity should be easy to use, accurate and reliable^(55)^. MUAC fulfil almost all characteristics; MUAC is simple to use since its measurement requires only a non-stretchable MUAC tape; MUAC measurement is easy to interpret since it does not require the use of an additional reference chart. Moreover, MUAC can easily be used by an illiterate person if it is colour-coded. Traffic light colours of red (obese), amber (overweight) and green (normal weight) may also be considered by non-numerate field workers in developing countries to facilitate screening^(17)^. The other important characteristic of ideal measurement is its accuracy in identifying overweight and obesity among children and adolescents. As we have observed in this meta-analysis, MUAC has high accuracy in identifying overweight and obesity. However, in this meta-analysis, all included articles compare the discriminatory ability of MUAC against BMI defined overweight and obesity. Even though BMI is highly correlated with percent body fat, it does not differentiate between lean mass and fat mass^(56)^. Only two studies have compared the discriminatory ability of MUAC with total body fat which was measured with bioelectrical impendency. A high level of discriminatory ability of MUAC has been reported by both studies^(18,44)^. None of the studies have assessed the discriminatory performance of MUAC with the four-compartment model, the known golden standard to measure total body fat. There is a need for future researches to explore the performance of MUAC to identify overweight and obesity, ideally as defined by the ‘golden standard’ measure of total body fat.

The major limitation of this meta-analysis was the presence of marked heterogenicity. First, a diagnostic threshold bias was identified as a cause of heterogeneity in the pooled results. In this meta-analysis, there was no consistent cut-off value. To overcome this limitation, we have used a hierarchal SROC curve that accounts for the threshold effect. Furthermore, we have conducted a sub-group analysis to reduce heterogeneity, we calculated measures of diagnostic performance according to age group, sex or weight status. Second, we have attempted to reach the corresponding authors of articles with insufficient data (prevalence, sample size by category) to be included in the meta-analysis, but without success. The main strength of this study was the rigorous statistical methods used to pool data across diagnostic accuracy studies. The other strength of this study is the comprehensive search strategy in several electronic databases.

## Conclusions

In conclusion, MUAC has high sensitivity and specificity compared with BMI defined overweight and obesity. However, there is no sufficient evidence on the performance of MUAC compared to gold standard measures of adiposity. There is a need for future studies to evaluate the diagnostic performance compared with the ‘golden standard’ measure of total body fat and evaluate the predictive value of the MUAC measurement for developing weight-related complications.

## Supplementary material

For supplementary material accompanying this paper visit https://doi.org/10.1017/S1368980022000143. click here to view supplementary material
