# Supplementary material for: The performance of mid-upper arm circumference for identifying children and adolescents with overweight and obesity: a systematic review and meta-analysis
Source: Public Health Nutr. 2022 Jan 17;25(3):607–16. doi: 10.1017/S1368980022000143 (PMC9991645; doi:10.1017/S1368980022000143)
Supplement: Supplementary file 1 [file S1368980022000143sup001.pdf]

PRISMA-DTA Checklist

Table S1

Section/topic	#	PRISMA-DTA Checklist Item	Reported on page #
**TITLE / ABSTRACT**			
Title	1	Identify the report as a systematic review (+/- meta-analysis) of diagnostic test accuracy (DTA) studies.	1
Abstract	2	Abstract: See PRISMA-DTA for abstracts.	
**INTRODUCTION**			
Rationale	3	Describe the rationale for the review in the context of what is already known.	3,4
Clinical role of index test	D1	State the scientific and clinical background, including the intended use and clinical role of the index test, and if applicable, the rationale for minimally acceptable test accuracy (or minimum difference in accuracy for comparative design).	3,4
Objectives	4	Provide an explicit statement of question(s) being addressed in terms of participants, index test(s), and target condition(s).	4
**METHODS**			
Protocol and registration	5	Indicate if a review protocol exists, if and where it can be accessed (e.g., Web address), and, if available, provide registration information including registration number.	5
Eligibility criteria	6	Specify study characteristics (participants, setting, index test(s), reference standard(s), target condition(s), and study design) and report characteristics (e.g., years considered, language, publication status) used as criteria for eligibility, giving rationale.	5,4
Information sources	7	Describe all information sources (e.g., databases with dates of coverage, contact with study authors to identify additional studies) in the search and date last searched.	5
Search	8	Present full search strategies for all electronic databases and other sources searched, including any limits used, such that they could be repeated.	supplementary file
Study selection	9	State the process for selecting studies (i.e., screening, eligibility, included in systematic review, and, if applicable, included in the meta-analysis).	5
Data collection process	10	Describe method of data extraction from reports (e.g., piloted forms, independently, in duplicate) and any processes for obtaining and confirming data from investigators.	6
Definitions for data extraction	11	Provide definitions used in data extraction and classifications of target condition(s), index test(s), reference standard(s) and other characteristics (e.g. study design, clinical setting).	6
Risk of bias and applicability	12	Describe methods used for assessing risk of bias in individual studies and concerns regarding the applicability to the review question.	6
Diagnostic accuracy measures	13	State the principal diagnostic accuracy measure(s) reported (e.g. sensitivity, specificity) and state the unit of assessment (e.g. per-patient, per-lesion).	6,7
Synthesis of results	14	Describe methods of handling data, combining results of studies and describing variability between studies. This could include, but is not limited to: a) handling of multiple definitions of target condition. b) handling of multiple	6,7

PRISMA-DTA Checklist

		thresholds of test positivity, c) handling multiple index test readers, d) handling of indeterminate test results, e) grouping and comparing tests, f) handling of different reference standards	

Section/topic	#	PRISMA-DTA Checklist Item	Reported on page #
Meta-analysis	D2	Report the statistical methods used for meta-analyses, if performed.	7
Additional analyses	16	Describe methods of additional analyses (e.g., sensitivity or subgroup analyses, meta-regression), if done, indicating which were pre-specified.	7
**RESULTS**			
Study selection	17	Provide numbers of studies screened, assessed for eligibility, included in the review (and included in meta-analysis, if applicable) with reasons for exclusions at each stage, ideally with a flow diagram.	8
Study characteristics	18	For each included study provide citations and present key characteristics including: a) participant characteristics (presentation, prior testing), b) clinical setting, c) study design, d) target condition definition, e) index test, f) reference standard, g) sample size, h) funding sources	8 and 18,25
Risk of bias and applicability	19	Present evaluation of risk of bias and concerns regarding applicability for each study.	Supplementary file
Results of individual studies	20	For each analysis in each study (e.g. unique combination of index test, reference standard, and positivity threshold) report 2x2 data (TP, FP, FN, TN) with estimates of diagnostic accuracy and confidence intervals, ideally with a forest or receiver operator characteristic (ROC) plot.	9,10
Synthesis of results	21	Describe test accuracy, including variability; if meta-analysis was done, include results and confidence intervals.	10 and 26
Additional analysis	23	Give results of additional analyses, if done (e.g., sensitivity or subgroup analyses, meta-regression; analysis of index test: failure rates, proportion of inconclusive results, adverse events).	10
**DISCUSSION**			
Summary of evidence	24	Summarize the main findings including the strength of evidence.	11,13
Limitations	25	Discuss limitations from included studies (e.g. risk of bias and concerns regarding applicability) and from the review process (e.g. incomplete retrieval of identified research).	12,13
Conclusions	26	Provide a general interpretation of the results in the context of other evidence. Discuss implications for future research and clinical practice (e.g. the intended use and clinical role of the index test).	11,13
**FUNDING**			
Funding	27	For the systematic review, describe the sources of funding and other support and the role of the funders.	14

Adapted From: McInnes MDF, Moher D, Thombs BD, McGrath TA, Bossuyt PM, The PRISMA-DTA Group (2018). Preferred Reporting Items for a Systematic Review and Meta-analysis of Diagnostic Test Accuracy Studies: The PRISMA-DTA Statement. JAMA. 2018 Jan 23;319(4):388-396. doi: 10.1001/jama.2017.19163.

For more information, visit: **www.prisma-statement.org**.

Table S2

Protocol method	Deviation from protocol method, with justification
We have planned to conduct a systematic search on PsycINFO.	However, we are not able to perform the systematic search PsycINFO bibliographic database due to lack of access to the database.<br><br>Type of deviation: omission
We were planning to assess the effect of risk of bias of included studies on diagnostic accuracy by performing a sensitivity analysis by excluding studies classified as having high or unclear risk of bias in at least one of the domains of QUADAS-2.	In the review we did not perform the sensitivity analysis due to lack of variation in the risk of bias and applicability domains throughout studies; all included articles have high risk of bias for reference standard under risk of bias domain.<br><br>Type of deviation: omission
We were planning to structure the search strategy from a set of search terms including index test, reference test, target condition and patient description.	In the review we have modified the search strategy to include only the following search terms: index test and target condition; after referring to reviewer's comments and Cochrane guideline(1) to diagnostic test accuracy reviews.<br><br>Type of deviation: modification

1. Macaskill P, Gatsonis C, Deeks J *et al.* (2010) Cochrane handbook for systematic reviews of diagnostic test accuracy. *Version 09 0 London: The Cochrane Collaboration* 83.

Table S3

Database	Search terms	
PubMed	Index test(s) set	("arm circumference"[Title/Abstract])
	Target condition set	((obese[Title/Abstract] OR obesity[Title/Abstract] OR overweight[Title/Abstract] OR "over weight"[Title/Abstract] OR "elevated body mass index"[Title/Abstract]))
Cochrane Library	Index test(s) set	(("arm circumference":ti,ab)
	Target condition set	((obese:ti,ab OR obesity:ti,ab OR overweight:ti,ab OR "over weight":ti,ab OR "elevated body mass index":ti,ab)))
Embase (Elsevier)	Index test(s) set	(("arm circumference":ti,ab)
	Target condition set	((obese:ti,ab OR obesity:ti,ab OR overweight:ti,ab OR "over weight":ti,ab OR "elevated body mass index":ti,ab)))
CINAHL (Ebsco)	Index test(s) set	(((TI "arm circumference" OR AB "arm circumference"))
	Target condition set	(((TI obese OR AB obese) OR (TI obesity OR AB obesity) OR (TI overweight OR AB overweight) OR (TI "over weight" OR AB "over weight") OR (TI "elevated body mass index" OR AB "elevated body mass index"))))
Web of Science	Index test(s) set	(("arm circumference")
	Target condition set	((obese OR obesity OR overweight OR "over weight" OR "elevated body mass index")))
Scopus (advanced search)	Index test(s) set	(("arm circumference")
	Target condition set	((obese OR obesity OR overweight OR "over weight" OR "elevated body mass index")))
Google scholar	Index test(s) set	(("arm circumference")
	Target condition set	((obese OR obesity OR overweight OR "over weight" OR "elevated body mass index")))

Table S4

ID	Primary author	year of publication	Location	Funder	Study design	Total sample size	Sample size / Sub group	Gender	Age (Years)	Index tests (MUAC)	Reference standard	Growth charts	overweight or obesity	Dx criteria according to reference standard	Dx criteria according to MUAC	Prevalence according to reference standard	AUC	Sensitivity	Specificity	LR+	LR-	Youden index	TP	FP	TN	FN
1	Lu et.al	2013	China	N/A	cross sectional	2847	208	Boys	7	MUAC	BMI	The 2004 Group of China Obesity Task Force	overweight	BMI ≥ 85th percentiles	18.9	0.18	0.934	0.877	0.89	7.97	0.13		33	19	152	5
							267	Boys	8	MUAC	BMI	The 2004 Group of China Obesity Task Force	overweight	BMI ≥ 85th percentiles	19.6	0.18	0.965	0.902	0.915	10.61	0.1		43	19	200	5
							264	Boys	9	MUAC	BMI	The 2004 Group of China Obesity Task Force	overweight	BMI ≥ 85th percentiles	21.1	0.18	0.967	0.871	0.957	20.25	0.13		41	9	207	6
							271	Boys	10	MUAC	BMI	The 2004 Group of China Obesity Task Force	overweight	BMI ≥ 85th percentiles	21.9	0.18	0.963	0.892	0.909	9.8	0.11		44	20	202	5
							277	Boys	11	MUAC	BMI	The 2004 Group of China Obesity Task Force	overweight	BMI ≥ 85th percentiles	22.6	0.18	0.94	0.825	0.93	11.78	0.18		41	16	211	9
							188	Boys	12	MUAC	BMI	The 2004 Group of China Obesity Task Force	overweight	BMI ≥ 85th percentiles	23.4	0.18	0.971	0.892	0.914	10.37	0.11		30	13	141	4
							184	Girls	7	MUAC	BMI	The 2004 Group of China Obesity Task Force	overweight	BMI ≥ 85th percentiles	18.9	0.12	0.943	0.906	0.817	4.95	0.11		20	30	133	2
							237	Girls	8	MUAC	BMI	The 2004 Group of China Obesity Task Force	overweight	BMI ≥ 85th percentiles	19.6	0.12	0.975	0.945	0.885	8.21	0.06		26	24	185	2

Table S4

ID	Primary author	year of publication	Location	Funder	Study design	Total sample size	Sample size / Sub group	Gender	Age (Years)	Index tests (MUAC)	Reference standard	Growth charts	overweight or obesity	Dx criteria according to reference standard	Dx criteria according to MUAC	Prevalence according to reference standard	AUC	Sensitivity	Specificity	LR+	LR-	Youden index	TP	FP	TN	FN
							246	Girls	9	MUAC	BMI	The 2004 Group of China Obesity Task Force	overweight	BMI ≥ 85th percentiles	20.4	0.12	0.956	0.942	0.881	7.91	0.06		27	26	191	2
							284	Girls	10	MUAC	BMI	The 2004 Group of China Obesity Task Force	overweight	BMI ≥ 85th percentiles	21.9	0.12	0.959	0.921	0.897	8.94	0.08		31	26	225	3
							238	Girls	11	MUAC	BMI	The 2004 Group of China Obesity Task Force	overweight	BMI ≥ 85th percentiles	22.6	0.12	0.944	0.836	0.932	12.69	0.17		23	14	196	5
							183	Girls	12	MUAC	BMI	The 2004 Group of China Obesity Task Force	overweight	BMI ≥ 85th percentiles	23.4	0.12	0.961	0.918	0.94	15.3	0.08		20	10	152	2
2	Rerksuppaphol et.al	2013	Thailand	This study was supported by the Faculty of Medicine, Srinakharinwirot University, Thailand.	cross sectional	3,618	360	Boys	7	MUAC	BMI	WHO BMI Z score	obesity	WHO >2 + SD	18.9	0.21	0.952	0.893	0.909	9.81	0.11		67	26	259	8
							303	Boys	8	MUAC	BMI	WHO BMI Z score	obesity	WHO >2 + SD	20.4	0.25	0.984	0.931	0.943	16.3	0.07		70	13	215	5
							307	Boys	9	MUAC	BMI	WHO BMI Z score	obesity	WHO >2 + SD	21.3	0.24	0.981	0.948	0.92	11.8	0.05		71	19	213	4
							290	Boys	10	MUAC	BMI	WHO BMI Z score	obesity	WHO >2 + SD	22.4	0.26	0.982	0.957	0.92	12.2	0.04		72	17	198	3
							318	Boys	11	MUAC	BMI	WHO BMI Z score	obesity	WHO >2 + SD	23.3	0.24	0.972	0.928	0.932	13.6	0.07		70	17	226	5
							252	Boys	12	MUAC	BMI	WHO BMI Z score	obesity	WHO >2 + SD	25.5	0.3	0.991	0.947	0.972	33.8	0.05		71	5	172	4

Table S4

ID	Primary author	year of publication	Location	Funder	Study design	Total sample size	Sample size / Sub group	Gender	Age (Years)	Index tests (MUAC)	Reference standard	Growth charts	overweight or obesity	Dx criteria according to reference standard	Dx criteria according to MUAC	Prevalence according to reference standard	AUC	Sensitivity	Specificity	LR+	LR-	Youden index	TP	FP	TN	FN
							310	Girls	7	MUAC	BMI	WHO BMI Z score	obesity	WHO >2 + SD	19.8	0.24	0.93	0.875	0.937	13.88	0.13		66	15	220	9
							326	Girls	8	MUAC	BMI	WHO BMI Z score	obesity	WHO >2 + SD	21.9	0.23	0.99	0.949	0.893	55.82	0.05		71	27	224	4
							298	Girls	9	MUAC	BMI	WHO BMI Z score	obesity	WHO >2 + SD	22.4	0.25	0.964	0.904	0.931	13.1	0.1		68	15	208	7
							298	Girls	10	MUAC	BMI	WHO BMI Z score	obesity	WHO >2 + SD	22.7	0.25	0.917	0.833	0.943	14.61	0.17		62	13	210	13
							330	Girls	11	MUAC	BMI	WHO BMI Z score	obesity	WHO >2 + SD	24.4	0.23	0.946	0.818	0.939	13.4	0.19		61	16	239	14
							226	Girls	12	MUAC	BMI	WHO BMI Z score	obesity	WHO >2 + SD	25.4	0.33	0.929	0.853	0.937	13.54	0.14		64	10	141	11
							285	Boys	7	MUAC	BMI	WHO BMI Z score	overweight	WHO >1 + SD	17.2	0.26	0.883	0.8	0.854	5.48	0.23		60	31	179	15
							255	Boys	8	MUAC	BMI	WHO BMI Z score	overweight	WHO >1 + SD	18.8	0.29	0.923	0.824	0.924	10.8	0.19		62	14	166	13
							249	Boys	9	MUAC	BMI	WHO BMI Z score	overweight	WHO >1 + SD	19.1	0.3	0.931	0.727	0.912	8.26	0.3		55	15	159	20
							243	Boys	10	MUAC	BMI	WHO BMI Z score	overweight	WHO >1 + SD	20.3	0.31	0.965	0.905	0.92	11.31	0.1		68	13	155	7
							249	Boys	11	MUAC	BMI	WHO BMI Z score	overweight	WHO >1 + SD	21.4	0.3	0.942	0.804	0.921	10.18	0.21		60	14	160	15

Table S4

ID	Primary author	year of publication	Location	Funder	Study design	Total sample size	Sample size / Sub group	Gender	Age (Years)	Index tests (MUAC)	Reference standard	Growth charts	overweight or obesity	Dx criteria according to reference standard	Dx criteria according to MUAC	Prevalence according to reference standard	AUC	Sensitivity	Specificity	LR+	LR-	Youden index	TP	FP	TN	FN
							214	Boys	12	MUAC	BMI	WHO BMI Z score	overweight	WHO >1 + SD	22.4	0.35	0.956	0.903	0.912	10.26	0.11		68	12	127	7
							310	Girls	7	MUAC	BMI	WHO BMI Z score	overweight	WHO >1 + SD	18	0.24	0.905	0.735	0.877	5.98	0.3		55	29	206	20
							326	Girls	8	MUAC	BMI	WHO BMI Z score	overweight	WHO >1 + SD	18.9	0.23	0.929	0.825	0.903	8.51	0.19		62	24	227	13
							298	Girls	9	MUAC	BMI	WHO BMI Z score	overweight	WHO >1 + SD	19.9	0.25	0.927	0.822	0.9	8.22	0.2		62	22	201	13
							298	Girls	10	MUAC	BMI	WHO BMI Z score	overweight	WHO >1 + SD	20.4	0.25	0.931	0.81	0.9	8.1	0.21		61	22	201	14
							330	Girls	11	MUAC	BMI	WHO BMI Z score	overweight	WHO >1 + SD	21.8	0.23	0.929	0.804	0.925	10.72	0.21		60	19	236	15
							226	Girls	12	MUAC	BMI	WHO BMI Z score	overweight	WHO >1 + SD	23.2	0.33	0.93	0.824	0.956	18.73	0.18		62	7	144	13
3	Asif et.al	2016	Pakistan	N/A	cross sectional	7,921	4,021	Boys	5	MUAC	BMI	WHO BMI Z score	overweight	BMI>+1SD	16.76		0.781	0.056	0.9	5.6	0.49					
								Boys	6	MUAC	BMI	WHO BMI Z score	overweight	BMI>+1SD	16.76		0.852	0.74	0.85	4.93	0.31					
								Boys	7	MUAC	BMI	WHO BMI Z score	overweight	BMI>+1SD	17.52		0.807	0.67	0.84	4.18	0.39					
								Boys	8	MUAC	BMI	WHO BMI Z score	overweight	BMI>+1SD	17.65		0.878	0.84	0.76	3.5	0.21					

Table S4

ID	Primary author	year of publication	Location	Funder	Study design	Total sample size	Sample size / Sub group	Gender	Age (Years)	Index tests (MUAC)	Reference standard	Growth charts	overweight or obesity	Dx criteria according to reference standard	Dx criteria according to MUAC	Prevalence according to reference standard	AUC	Sensitivity	Specificity	LR+	LR-	Youden index	TP	FP	TN	FN
								Boys	9	MUAC	BMI	WHO BMI Z score	overweight	BMI>+1SD	18.41		0.909	0.85	0.92	10.62	0.16					
								Boys	10	MUAC	BMI	WHO BMI Z score	overweight	BMI>+1SD	19.17		0.762	0.55	0.94	9.16	0.48					
								Boys	11	MUAC	BMI	WHO BMI Z score	overweight	BMI>+1SD	19.43		0.759	0.67	0.89	6.09	0.37					
								Boys	12	MUAC	BMI	WHO BMI Z score	overweight	BMI>+1SD	19.17		0.843	0.81	0.79	3.85	0.24					
								Boys	13	MUAC	BMI	WHO BMI Z score	overweight	BMI>+1SD	20.7		0.849	0.79	0.9	7.9	0.23					
								Boys	14	MUAC	BMI	WHO BMI Z score	overweight	BMI>+1SD	22.73		0.849	0.69	0.89	6.27	0.35					
								Boys	14-Jun	MUAC	BMI	WHO BMI Z score	overweight	BMI>+1SD	19.17	0.2	0.791	0.66	0.82	3.66	0.41		520	582	2651	268
							3,900	Girls	5	MUAC	BMI	WHO BMI Z score	overweight	BMI>+1SD	16.38		0.744	0.57	0.84	3.56	0.51					
								Girls	6	MUAC	BMI	WHO BMI Z score	overweight	BMI>+1SD	16.38		0.788	0.73	0.76	3.04	0.36					
								Girls	7	MUAC	BMI	WHO BMI Z score	overweight	BMI>+1SD	17.52		0.884	0.8	0.86	5.71	0.23					
								Girls	8	MUAC	BMI	WHO BMI Z score	overweight	BMI>+1SD	17.9		0.912	0.78	0.93	11.14	0.24					

Table S4

ID	Primary author	year of publication	Location	Funder	Study design	Total sample size	Sample size / Sub group	Gender	Age (Years)	Index tests (MUAC)	Reference standard	Growth charts	overweight or obesity	Dx criteria according to reference standard	Dx criteria according to MUAC	Prevalence according to reference standard	AUC	Sensitivity	Specificity	LR+	LR-	Youden index	TP	FP	TN	FN
								Girls	9	MUAC	BMI	WHO BMI Z score	overweight	BMI>+1SD	17.9		0.799	0.63	0.86	4.5	0.43					
								Girls	10	MUAC	BMI	WHO BMI Z score	overweight	BMI>+1SD	18.16		0.733	0.58	0.81	3.05	0.52					
								Girls	11	MUAC	BMI	WHO BMI Z score	overweight	BMI>+1SD	19.68		0.857	0.72	0.86	5.14	0.33					
								Girls	12	MUAC	BMI	WHO BMI Z score	overweight	BMI>+1SD	20.57		0.829	0.58	0.94	9.67	0.45					
								Girls	13	MUAC	BMI	WHO BMI Z score	overweight	BMI>+1SD	19.17		0.801	0.88	0.59	2.14	0.2					
								Girls	14	MUAC	BMI	WHO BMI Z score	overweight	BMI>+1SD	19.68		0.795	0.9	0.55	2	0.18					
								Girls	14-Jun	MUAC	BMI	WHO BMI Z score	overweight	BMI>+1SD	17.9	0.19	0.782	0.7	0.75	2.8	0.4		516	791	2372	221
4	Craig et.al	2013	South Africa	Yorkhill Children's Foundation	cross sectional	978	235	Girls	5–9	MUAC	BMI	WHO BMI Z score	overweight	BMI>+1SD			0.96	0.971	0.791	4.65	0.037					
								Girls	5-9	MUAC	BMI	WHO BMI Z score	overweight	BMI>+1SD				0.941	0.881	7.88	0.066					
							246	Boys	5-9	MUAC	BMI	WHO BMI Z score	overweight	BMI>+1SD			0.9	0.68	0.892	6.32	0.3586					
								Boys	5-9	MUAC	BMI	WHO BMI Z score	overweight	BMI>+1SD				0.64	0.933	9.51	0.386					

Table S4

ID	Primary author	year of publication	Location	Funder	Study design	Total sample size	Sample size / Sub group	Gender	Age (Years)	Index tests (MUAC)	Reference standard	Growth charts	overweight or obesity	Dx criteria according to reference standard	Dx criteria according to MUAC	Prevalence according to reference standard	AUC	Sensitivity	Specificity	LR+	LR-	Youden index	TP	FP	TN	FN
							269	Girls	10-14	MUAC	BMI	WHO BMI Z score	overweight	BMI>+1SD			0.94	0.929	0.78	4.22	0.09					
								Girls	10-14	MUAC	BMI	WHO BMI Z score	overweight	BMI>+1SD				0.881	0.811	4.65	0.15					
							228	Boys	10-14	MUAC	BMI	WHO BMI Z score	overweight	BMI>+1SD			0.94	0.952	0.899	9.39	0.05					
								Boys	10-14	MUAC	BMI	WHO BMI Z score	overweight	BMI>+1SD				0.762	0.762	13.14	0.25					
							235	Girls	5-9	MUAC	BIA	McCarthy 2006 body fat reference curves	overweight	> 85th percentile				0.638	0.904	6.67	0.4					
								Girls	5-9	MUAC	BIA	McCarthy 2006 body fat reference curves	overweight	> 85th percentile				0.575	0.872	10.8	0.45					
							246	Boys	5-9	MUAC	BIA	McCarthy 2006 body fat reference curves	overweight	> 85th percentile				0.279	0.756	1.95	0.84					
								Boys	5-9	MUAC	BIA	McCarthy 2006 body fat reference curves	overweight	> 85th percentile				0.256	0.793	2.73	0.82					

Table S4

ID	Primary author	year of publication	Location	Funder	Study design	Total sample size	Sample size / Sub group	Gender	Age (Years)	Index tests (MUAC)	Reference standard	Growth charts	overweight or obesity	Dx criteria according to reference standard	Dx criteria according to MUAC	Prevalence according to reference standard	AUC	Sensitivity	Specificity	LR+	LR-	Youden index	TP	FP	TN	FN
							269	Girls	10-14	MUAC	BIA	McCarthy 2006 body fat reference curves	overweight	> 85th percentile				1	0.725	3.23	0					
								Girls	10-14	MUAC	BIA	McCarthy 2006 body fat reference curves	overweight	> 85th percentile				1	0.799	4.43	0					
							228	Boys	10-14	MUAC	BIA	McCarthy 2006 body fat reference curves	overweight	> 85th percentile				0.923	0.939	15.27	0.08					
								Boys	10-14	MUAC	BIA	McCarthy 2006 body fat reference curves	overweight	> 85th percentile				0.923	0.947	18.04	0.08					
5	Chaput et.al	2016	12 countries	The Coca≥Cola Company	cross sectional	7337	3408	Boys	9–11 years	MUAC	BMI	WHO BMI Z score	obesity	BMI> +2SD	24.6	0.0155	0.98	0.95	0.92			0.87	50	268	3087	3
							3929	Girls	9-11	MUAC	BMI	WHO BMI Z score	obesity	BMI> +2SD	25.2	0.101	0.97	0.94	0.9			0.84	373	353	3179	24
							7337	Boys and girls	9-11	MUAC	BMI	WHO BMI Z score	obesity	BMI> +2SD	24.8	0.126073	0.97	0.95	0.9			0.84	879	641	5771	46
6	Jaiswal et.al	2017	India		cross sectional	875	222	Males	5-9 years	MUAC	BMI	IAP growth charts	overweight	BMI falling between 23rd and	18.8		0.93	0.934	.95.1	19.44	0.06					

Table S4

ID	Primary author	year of publication	Location	Funder	Study design	Total sample size	Sample size / Sub group	Gender	Age (Years)	Index tests (MUAC)	Reference standard	Growth charts	overweight or obesity	Dx criteria according to reference standard	Dx criteria according to MUAC	Prevalence according to reference standard	AUC	Sensitivity	Specificity	LR+	LR-	Youden index	TP	FP	TN	FN
														27th adult equivalent												
						436	221	Females	5-9 years	MUAC	BMI	IAP growth charts	overweight	BMI falling between 23rd and 27th adult equivalent	19.4		0.92	.91.6	.97.3	34.83	0.08					
						439	214	Males	10-14 years	MUAC	BMI	IAP growth charts	overweight	BMI falling between 23rd and 27th adult equivalent	23		0.98	0.1	0.9722	36	0					
							218	Females	10-14 years	MUAC	BMI	IAP growth charts	overweight	BMI falling between 23rd and 27th adult equivalent	23.3		0.98	0.909	1	36	0.09					
7	Talma et.al	2018	Dutch	The Dutch Ministry of Health,Welfare and Sport	cross sectional	6167		Boys	2-5 years	MUAC	BMI	International Obesity Task Force	overweight	BMI ≥ 85th percentiles	>1.3 SDS		0.9	0.518	0.938							
								Boys	6-11 years	MUAC	BMI	International Obesity Task Force	overweight	BMI ≥ 85th percentiles	>1.3 SDS		0.94	0.953	0.714							
								Boys	12-18 years	MUAC	BMI	International Obesity Task Force	overweight	BMI ≥ 85th percentiles	>1.3 SDS		0.94	0.923	0.823							

Table S4

ID	Primary author	year of publication	Location	Funder	Study design	Total sample size	Sample size / Sub group	Gender	Age (Years)	Index tests (MUAC)	Reference standard	Growth charts	overweight or obesity	Dx criteria according to reference standard	Dx criteria according to MUAC	Prevalence according to reference standard	AUC	Sensitivity	Specificity	LR+	LR-	Youden index	TP	FP	TN	FN
								Girls	2-5 years	MUAC	BMI	International Obesity Task Force	overweight	BMI ≥ 85th percentiles	>1.3 SDS		0.88	0.541	0.923							
								Girls	6-11 years	MUAC	BMI	International Obesity Task Force	overweight	BMI ≥ 85th percentiles	>1.3 SDS		0.94	0.904	0.803							
								Girls	12-18 years	MUAC	BMI	International Obesity Task Force	overweight	BMI ≥ 85th percentiles	>1.3 SDS		0.93	0.755	0.909							
8	Maz≥c≥o≥lu et.al	2006	Turkey	N/A	cross sectional	5358	2621 boys & 2737 girls	Boys	6	MUAC	BMI	International Obesity Task Force	overweight	BMI ≥ 85th percentiles	18.1		0.684	0.5	0.889							
								Boys	7	MUAC	BMI	International Obesity Task Force	overweight	BMI ≥ 85th percentiles	18.1		0.697	0.578	0.798							
								Boys	8	MUAC	BMI	International Obesity Task Force	overweight	BMI ≥ 85th percentiles	18.9		0.791	0.638	0.799							
								Boys	9	MUAC	BMI	International Obesity Task Force	overweight	BMI ≥ 85th percentiles	20.4		0.873	0.684	0.976							
								Boys	10	MUAC	BMI	International Obesity Task Force	overweight	BMI ≥ 85th percentiles	19.9		0.939	0.971	0.769							

Table S4

ID	Primary author	year of publication	Location	Funder	Study design	Total sample size	Sample size / Sub group	Gender	Age (Years)	Index tests (MUAC)	Reference standard	Growth charts	overweight or obesity	Dx criteria according to reference standard	Dx criteria according to MUAC	Prevalence according to reference standard	AUC	Sensitivity	Specificity	LR+	LR-	Youden index	TP	FP	TN	FN
								Boys	11	MUAC	BMI	International Obesity Task Force	overweight	BMI ≥ 85th percentiles	21.9		0.891	0.738	0.888							
								Boys	12	MUAC	BMI	International Obesity Task Force	overweight	BMI ≥ 85th percentiles	21.9		0.919	0.738	0.888							
								Boys	13	MUAC	BMI	International Obesity Task Force	overweight	BMI ≥ 85th percentiles	22.6		0.919	0.9	0.825							
								Boys	14	MUAC	BMI	International Obesity Task Force	overweight	BMI ≥ 85th percentiles	22.8		0.874	0.868	0.764							
								Boys	15	MUAC	BMI	International Obesity Task Force	overweight	BMI ≥ 85th percentiles	24.9		0.852	0.73	0.837							
								Boys	16	MUAC	BMI	International Obesity Task Force	overweight	BMI ≥ 85th percentiles	24.2		0.884	0.841	0.782							
								Boys	17	MUAC	BMI	International Obesity Task Force	overweight	BMI ≥ 85th percentiles	25.7		0.898	0.87	0.878							
								Girls	6	MUAC	BMI	International Obesity Task Force	overweight	BMI ≥ 85th percentiles	17.9		0.645	0.677	0.758							

Table S4

ID	Primary author	year of publication	Location	Funder	Study design	Total sample size	Sample size / Sub group	Gender	Age (Years)	Index tests (MUAC)	Reference standard	Growth charts	overweight or obesity	Dx criteria according to reference standard	Dx criteria according to MUAC	Prevalence according to reference standard	AUC	Sensitivity	Specificity	LR+	LR-	Youden index	TP	FP	TN	FN
								Girls	7	MUAC	BMI	International Obesity Task Force	overweight	BMI ≥ 85th percentiles	18.2		0.721	0.6	0.832							
								Girls	8	MUAC	BMI	International Obesity Task Force	overweight	BMI ≥ 85th percentiles	18.7		0.862	0.808	0.75							
								Girls	9	MUAC	BMI	International Obesity Task Force	overweight	BMI ≥ 85th percentiles	20.2		0.89	0.767	0.905							
								Girls	10	MUAC	BMI	International Obesity Task Force	overweight	BMI ≥ 85th percentiles	20.6		0.887	0.813	0.86							
								Girls	11	MUAC	BMI	International Obesity Task Force	overweight	BMI ≥ 85th percentiles	20.5		0.913	0.947	0.687							
								Girls	12	MUAC	BMI	International Obesity Task Force	overweight	BMI ≥ 85th percentiles	22.6		0.902	0.739	0.894							
								Girls	13	MUAC	BMI	International Obesity Task Force	overweight	BMI ≥ 85th percentiles	22.8		0.876	0.853	0.827							
								Girls	14	MUAC	BMI	International Obesity Task Force	overweight	BMI ≥ 85th percentiles	23.8		0.869	0.845	0.764							

Table S4

ID	Primary author	year of publication	Location	Funder	Study design	Total sample size	Sample size / Sub group	Gender	Age (Years)	Index tests (MUAC)	Reference standard	Growth charts	overweight or obesity	Dx criteria according to reference standard	Dx criteria according to MUAC	Prevalence according to reference standard	AUC	Sensitivity	Specificity	LR+	LR-	Youden index	TP	FP	TN	FN
								Girls	15	MUAC	BMI	International Obesity Task Force	overweight	BMI ≥ 85th percentiles	23.9		0.907	0.891	0.815							
								Girls	16	MUAC	BMI	International Obesity Task Force	overweight	BMI ≥ 85th percentiles	23.9		0.857	0.82	0.781							
								Girls	17	MUAC	BMI	International Obesity Task Force	overweight	BMI ≥ 85th percentiles	24.5		0.9131	0.857	0.869							
								Boys	6	MUAC	WC			WC ≥90thpercentilein	18.2		0.755	0.668	0.821							
								Boys	7	MUAC	WC			WC ≥90thpercentilein	19.8		0.744	0.526	0.936							
								Boys	8	MUAC	WC			WC ≥90thpercentilein	19.4		0.862	0.7	0.869							
								Boys	9	MUAC	WC			WC ≥90thpercentilein	20.9		0.926	0.87	0.915							
								Boys	10	MUAC	WC			WC ≥90thpercentilein	21.2		0.93	0.833	0.889							
								Boys	11	MUAC	WC			WC ≥90thpercentilein	22		0.939	0.882	0.868							
								Boys	12	MUAC	WC			WC ≥90thpercentilein	21.7		0.912	0.895	0.778							

Table S4

ID	Primary author	year of publication	Location	Funder	Study design	Total sample size	Sample size / Sub group	Gender	Age (Years)	Index tests (MUAC)	Reference standard	Growth charts	overweight or obesity	Dx criteria according to reference standard	Dx criteria according to MUAC	Prevalence according to reference standard	AUC	Sensitivity	Specificity	LR+	LR-	Youden index	TP	FP	TN	FN
								Boys	13	MUAC	WC			WC ≥90thpercentilein	23.5		0.972	1	0.883							
								Boys	14	MUAC	WC			WC ≥90thpercentilein	24.3		0.954	0.926	0.86							
								Boys	15	MUAC	WC			WC ≥90thpercentilein	25.3		0.91	0.814	0.892							
								Boys	16	MUAC	WC			WC ≥90thpercentilein	26		0.928	0.821	0.907							
								Boys	17	MUAC	WC			WC ≥90thpercentilein	26.5		0.859	0.722	0.924							
								Girls	6	MUAC	WC			WC ≥90thpercentilein	18		0.84	0.769	0.814							
								Girls	7	MUAC	WC			WC ≥90thpercentilein	17.9		0.883	1	0.633							
								Girls	8	MUAC	WC			WC ≥90thpercentilein	20.1		0.946	0.93	0.115							
								Girls	9	MUAC	WC			WC ≥90thpercentilein	20.3		0.909	0.882	0.871							
								Girls	10	MUAC	WC			WC ≥90thpercentilein	22.3		0.893	0.8	0.923							
								Girls	11	MUAC	WC			WC ≥90thpercentilein	22.9		0.894	0.722	0.894							

Table S4

ID	Primary author	year of publication	Location	Funder	Study design	Total sample size	Sample size / Sub group	Gender	Age (Years)	Index tests (MUAC)	Reference standard	Growth charts	overweight or obesity	Dx criteria according to reference standard	Dx criteria according to MUAC	Prevalence according to reference standard	AUC	Sensitivity	Specificity	LR+	LR-	Youden index	TP	FP	TN	FN
								Girls	12	MUAC	WC			WC ≥90thpercentilein	21.9		0.905	1	0.678							
								Girls	13	MUAC	WC			WC ≥90thpercentilein	23		0.93	0.95	0.852							
								Girls	14	MUAC	WC			WC ≥90thpercentilein	24.1		0.927	0.939	0.81							
								Girls	15	MUAC	WC			WC ≥90thpercentilein	23.9		0.919	0.951	0.778							
								Girls	16	MUAC	WC			WC ≥90thpercentilein	44.3		0.926	0.943	0.83							
								Girls	17	MUAC	WC			WC ≥90thpercentilein	25.7		0.887	0.692	0.906							
9	Ayu et al. (47) (2017)	2017	Indonesia	N/A	cross sectional	2,258	850	Boys	6-7	MUAC	BMI	2000 CDC BMI reference standard curves	overweight	BMI ≥ 85th	18.5	0.144504		0.892	0.783	4.1	0.15		110	158	569	13
							826	Girls	6-7	MUAC	BMI	2000 CDC BMI reference standard curves	overweight	BMI ≥ 85th	18.5	0.148843		0.866	0.783	3.99	0.17		106	153	550	16
							1677	Boys and girls	6-7	MUAC	BMI	2000 CDC BMI reference standard curves	overweight	BMI ≥ 85th	18.5	0.136949		0.881	0.783	4.05	0.15		202	314	1133	27
							1159	Boys	6-7	MUAC	BMI	2000 CDC BMI reference standard curves	obesity	BMI ≥ 95th percentile	19.5	0.07572		0.9325	0.869	6.64	0.77		82	140	931	6

Table S4

ID	Primary author	year of publication	Location	Funder	Study design	Total sample size	Sample size / Sub group	Gender	Age (Years)	Index tests (MUAC)	Reference standard	Growth charts	overweight or obesity	Dx criteria according to reference standard	Dx criteria according to MUAC	Prevalence according to reference standard	AUC	Sensitivity	Specificity	LR+	LR-	Youden index	TP	FP	TN	FN
							1099	Girls	6-7	MUAC	BMI	2000 CDC BMI reference standard curves	obesity	BMI ≥ 95th percentile	19.5	0.062595		0.851	0.862	6.15	0.17		59	142	888	10
							2258	Boys and girls	6-7	MUAC	BMI	2000 CDC BMI reference standard curves	obesity	BMI ≥ 95th percentile	19.5	0.069749		0.9015	0.8665	6.28	0.13		142	280	1820	16
							1413	Boys and girls	6-7	MUAC	BMI	2000 CDC BMI reference standard curves	overweight	BMI ≥ 85 - <95 percentile	18.5	0.074542		0.786	0.783	3.46	0.28		83	284	1024	23
							687	Boys	6-7	MUAC	BMI	2000 CDC BMI reference standard curves	overweight	BMI ≥ 85 - <95 percentile	18.5	0.28657		0.733	0.783	3.22	0.35		144	106	384	53
							726	Girls	6-7	MUAC	BMI	2000 CDC BMI reference standard curves	overweight	BMI ≥ 85 - <95 percentile	18.5	0.1085		0.83	0.783	3.8	0.21		65	140	507	13
10	Shinsugi et.al	2020	Sri Lanka	Japan Society for the Promotion of Science	cross sectional	528	528	Boys and girls	5–10 years	MUAC	BMI	WHO BMI Z score	overweight	BAZ > +1 SD	19.05	0.128	0.92	0.99	0.85				68	70	399	1
								Boys and girls	5–10 years	MUAC	BMI	WHO BMI Z score	obesity	BAZ > +2 SD	21.8		0.96	0.96	0.96				26	22	489	1
								Boys and girls	5–7 years	MUAC	BMI	WHO BMI Z score	overweight	BAZ > +1 SD	190.5		0.97	1	0.94				27	14	218	0

Table S4

ID	Primary author	year of publication	Location	Funder	Study design	Total sample size	Sample size / Sub group	Gender	Age (Years)	Index tests (MUAC)	Reference standard	Growth charts	overweight or obesity	Dx criteria according to reference standard	Dx criteria according to MUAC	Prevalence according to reference standard	AUC	Sensitivity	Specificity	LR+	LR-	Youden index	TP	FP	TN	FN
								Boys and girls	8–10 years	MUAC	BMI	WHO BMI Z score	overweight	BAZ > +1 SD	205.5		0.93	0.95	0.92				40	20	217	2
11	Dumith et.al	2011	Brazil	National Council for Scientific and Technological Development of the Ministry of Science	cross sectional	1,075	254	Boys	13-15 year	MUAC	BMI	WHO BMI Z score	overweight	BMI>+1SD		0.093403	0.8	0.714	0.73				17	62	168	7
							258	Boys	16-19 year	MUAC	BMI	WHO BMI Z score	overweight	BMI>+1SD		0.072206	0.67	0.579	0.569				11	103	136	8
							272	Girls	13-15 year	MUAC	BMI	WHO BMI Z score	overweight	BMI>+1SD		0.056836	0.93	0.84	0.891				13	28	229	2
							291	Girls	16-19 year	MUAC	BMI	WHO BMI Z score	overweight	BMI>+1SD		0.076552	0.93	0.757	0.878				17	33	236	5
12	Otitoola et.al	2020	South Africa	N/A	cross sectional	211		Girls	6–9 years	MUAC	BMI	WHO BMI Z score	overweight	BMI >+1SD			0.763									
								Boys	6-9	MUAC	BMI	WHO BMI Z score	overweight	BMI >+1SD			0.944									
								Girls	10-19	MUAC	BMI	WHO BMI Z score	overweight	BMI >+1SD			0.934									
								Boys	10-19	MUAC	BMI	WHO BMI Z score	overweight	BMI >+1SD			0.899									
								Total	6-19	MUAC	BMI	WHO BMI Z score	overweight	BMI >+1SD			0.795									

Table S4

ID	Primary author	year of publication	Location	Funder	Study design	Total sample size	Sample size / Sub group	Gender	Age (Years)	Index tests (MUAC)	Reference standard	Growth charts	overweight or obesity	Dx criteria according to reference standard	Dx criteria according to MUAC	Prevalence according to reference standard	AUC	Sensitivity	Specificity	LR+	LR-	Youden index	TP	FP	TN	FN
13	Okosun OA et.al	2019	Nigeria	N/A	cross sectional	920	205	Boys and girls	5-9 years	MUAC	BMI	WHO BMI Z score	obesity	BAZ > +2 SD		0.493194		0.72	0.47				73	55	49	28
							526	Boys and girls	10-14 years	MUAC	BMI	WHO BMI Z score	obesity	BAZ > +2 SD		0.046154		0.91	0.88				22	60	442	2
							189	Boys and girls	15-18 years	MUAC	BMI	WHO BMI Z score	obesity	BAZ > +2 SD		0.041096		0.73	0.75				6	45	136	2
							98	Boys	5-9 years	MUAC	BMI	WHO BMI Z score	obesity	BAZ > +2 SD		0.593103		0.58	0.2				34	32	8	24
							232	Boys	10-14 years	MUAC	BMI	WHO BMI Z score	obesity	BAZ > +2 SD		0.070787		0.89	0.82				15	39	177	2
							74	Boys	15-18 years	MUAC	BMI	WHO BMI Z score	obesity	BAZ > +2 SD		0		0.36	0				0	74	0	0
							108	Girls	5-9 years	MUAC	BMI	WHO BMI Z score	obesity	BAZ > +2 SD		0.293012		0.83	0.68				26	24	52	5
							294	Girls	10-14 years	MUAC	BMI	WHO BMI Z score	obesity	BAZ > +2 SD		0.028936		0.94	0.92				8	23	263	1
							115	Girls	15-18 years	MUAC	BMI	WHO BMI Z score	obesity	BAZ > +2 SD		0.032308		0.78	0.86				3	16	96	1
14	ASIF et.al	2018	Pakistan	N/A	cross sectional	4962		Boys	12	MUAC	BMI	CDC growth charts	overweight	≥ 85th percentile	19.43		0.88	0.91	0.74	3.53	-0.23					

Table S4

ID	Primary author	year of publication	Location	Funder	Study design	Total sample size	Sample size / Sub group	Gender	Age (Years)	Index tests (MUAC)	Reference standard	Growth charts	overweight or obesity	Dx criteria according to reference standard	Dx criteria according to MUAC	Prevalence according to reference standard	AUC	Sensitivity	Specificity	LR+	LR-	Youden index	TP	FP	TN	FN
								Boys	13	MUAC	BMI	CDC growth charts	overweight	≥ 85th percentile	20.7		0.88	0.86	0.83	5.18	-0.03					
								Boys	14	MUAC	BMI	CDC growth charts	overweight	≥ 85th percentile	21.73		0.86	0.77	0.85	5.21	0.09					
								Boys	15	MUAC	BMI	CDC growth charts	overweight	≥ 85th percentile	22.48		0.84	0.65	0.92	7.79	0.28					
								Boys	16	MUAC	BMI	CDC growth charts	overweight	≥ 85th percentile	22.73		0.83	0.85	0.65	2.41	-0.31					
								Boys	17	MUAC	BMI	CDC growth charts	overweight	≥ 85th percentile	22.98		0.84	0.74	0.78	3.39	0.05					
								Boys	18	MUAC	BMI	CDC growth charts	overweight	≥ 85th percentile	23.11		0.77	0.84	0.62	2.24	-0.34					
								Girls	12	MUAC	BMI	CDC growth charts	overweight	≥ 85th percentile	19.56		0.85	0.83	0.74	3.24	-0.12					
								Girls	13	MUAC	BMI	CDC growth charts	overweight	≥ 85th percentile	21.08		0.82	0.69	0.83	4.09	0.17					
								Girls	14	MUAC	BMI	CDC growth charts	overweight	≥ 85th percentile	20.57		0.79	0.66	0.79	3.08	0.17					
								Girls	15	MUAC	BMI	CDC growth charts	overweight	≥ 85th percentile	20.58		0.7	0.72	0.65	2.09	-0.09					
								Girls	16	MUAC	BMI	CDC growth charts	overweight	≥ 85th percentile	21.71		0.7	0.68	0.7	2.26	0.03					

Table S4

ID	Primary author	year of publication	Location	Funder	Study design	Total sample size	Sample size / Sub group	Gender	Age (Years)	Index tests (MUAC)	Reference standard	Growth charts	overweight or obesity	Dx criteria according to reference standard	Dx criteria according to MUAC	Prevalence according to reference standard	AUC	Sensitivity	Specificity	LR+	LR-	Youden index	TP	FP	TN	FN
								Girls	17	MUAC	BMI	CDC growth charts	overweight	≥ 85th percentile	23.62		0.7	0.47	0.85	3.07	0.45					
								Girls	18	MUAC	BMI	CDC growth charts	overweight	≥ 85th percentile	24.76		0.81	0.78	0.75	3.17	-0.04					
						1,090	1,090	Boys and girls	Pre school children	MUAC for age	BMI			Z score greater than 2 for the weight	0.7 (z score)			0.765	0.779							
										MUAC for hight	BMI				0.6 (z score)			0.794	0.776							
15	Oriaifo et.al	2019	Nigeria	N/A	cross sectional	1,067	43	Boys	6	MUAC	BIA	Mc Carthy reference	overweight	≥ 85th percentile	18.75		0.86	0.8	0.862	5.8	0.23					
							41	Boys	7	MUAC	BIA	Mc Carthy reference	overweight	≥ 85th percentile	19.75		0.83	0.6	0.944	10.71	0.42					
							42	Boys	8	MUAC	BIA	Mc Carthy reference	overweight	≥ 85th percentile	22.75		0.99	1	0.975	40	0					
							40	Boys	9	MUAC	BIA	Mc Carthy reference	overweight	≥ 85th percentile			N/A									
							43	Boys	10	MUAC	BIA	Mc Carthy reference	overweight	≥ 85th percentile	24.75		1	1	1	0	0					
							40	Boys	11	MUAC	BIA	Mc Carthy reference	overweight	≥ 85th percentile	24.5		1	1	1	0	0					

Table S4

ID	Primary author	year of publication	Location	Funder	Study design	Total sample size	Sample size / Sub group	Gender	Age (Years)	Index tests (MUAC)	Reference standard	Growth charts	overweight or obesity	Dx criteria according to reference standard	Dx criteria according to MUAC	Prevalence according to reference standard	AUC	Sensitivity	Specificity	LR+	LR-	Youden index	TP	FP	TN	FN
							38	Boys	12	MUAC	BIA	Mc Carthy reference	overweight	≥ 85th percentile	24.25		0.81	0.667	0.857	4.66	0.39					
							42	Boys	13	MUAC	BIA	Mc Carthy reference	overweight	≥ 85th percentile	25.5		0.89	1	0.878	8.2	0					
							40	Boys	14	MUAC	BIA	Mc Carthy reference	overweight	≥ 85th percentile	25.75		0.85	1	0.757	4.12	0					
							46	Boys	15	MUAC	BIA	Mc Carthy reference	overweight	≥ 85th percentile	30		0.79	0.8	0.854	5.48	0.23					
							39	Boys	16	MUAC	BIA	Mc Carthy reference	overweight	≥ 85th percentile	26.25		0.62	0.8	0.529	1.7	0.38					
							41	Boys	17	MUAC	BIA	Mc Carthy reference	overweight	≥ 85th percentile	27.75		0.66	0.8	0.556	1.8	0.36					
							43	Boys	18	MUAC	BIA	Mc Carthy reference	overweight	≥ 85th percentile	31.5		0.97	1	0.946	18.52	0					
							40	Girls	6	MUAC	BIA	Mc Carthy reference	overweight	≥ 85th percentile	20.75		0.99	1	94.4	17.86	0					
							41	Girls	7	MUAC	BIA	Mc Carthy reference	overweight	≥ 85th percentile	21.75		0.8	66.7	1	0	0.33					
							40	Girls	8	MUAC	BIA	Mc Carthy reference	overweight	≥ 85th percentile	22		0.98	1	0.971	34.48	0					
							42	Girls	9	MUAC	BIA	Mc Carthy reference	overweight	≥ 85th percentile	24		1	1	1	0	0					

Table S4

ID	Primary author	year of publication	Location	Funder	Study design	Total sample size	Sample size / Sub group	Gender	Age (Years)	Index tests (MUAC)	Reference standard	Growth charts	overweight or obesity	Dx criteria according to reference standard	Dx criteria according to MUAC	Prevalence according to reference standard	AUC	Sensitivity	Specificity	LR+	LR-	Youden index	TP	FP	TN	FN
							39	Girls	10	MUAC	BIA	Mc Carthy reference	overweight	≥ 85th percentile	22.25		0.92	0.8	0.966	23.53	0.21					
							42	Girls	11	MUAC	BIA	Mc Carthy reference	overweight	≥ 85th percentile	26.75		0.98	1	0.973	37.04	0					
							43	Girls	12	MUAC	BIA	Mc Carthy reference	overweight	≥ 85th percentile	27.75		0.99	1	0.974	38.46	0					
							40	Girls	13	MUAC	BIA	Mc Carthy reference	overweight	≥ 85th percentile	25.75		0.85	0.75	0.722	2.7	0.35					
							42	Girls	14	MUAC	BIA	Mc Carthy reference	overweight	≥ 85th percentile	25.8		0.94	1	0.769	4.33	0					
							38	Girls	15	MUAC	BIA	Mc Carthy reference	overweight	≥ 85th percentile	27.25		0.87	0.84	0.8	4.23	0.19					
							43	Girls	16	MUAC	BIA	Mc Carthy reference	overweight	≥ 85th percentile	28.75		0.78	0.71	0.722	2.57	0.4					
							40	Girls	17	MUAC	BIA	Mc Carthy reference	overweight	≥ 85th percentile	29		0.77	0.6	0.767	2.58	0.52					
							39	Girls	18	MUAC	BIA	Mc Carthy reference	overweight	≥ 85th percentile	26.25		0.88	0.875	0.8	4.38	0.16					
16	Sisay et.al	2020	Ethiopia	Addis Ababa University	cross sectional	851	456	Boys	15-19 years	MUAC	BMI	WHO BMI Z score	overweight	BAZ > +1 SD	27.75	11.2		0.941	0.891	8.7	0.07		48	44	361	3
							395	Girls	15-19 years	MUAC	BMI	WHO BMI Z score	overweight	BAZ > +1 SD	27.9	18.2		0.903	0.907	9.7	0.11		65	30	293	7

Table S4

ID	Primary author	year of publication	Location	Funder	Study design	Total sample size	Sample size / Sub group	Gender	Age (Years)	Index tests (MUAC)	Reference standard	Growth charts	overweight or obesity	Dx criteria according to reference standard	Dx criteria according to MUAC	Prevalence according to reference standard	AUC	Sensitivity	Specificity	LR+	LR-	Youden index	TP	FP	TN	FN
							851	Boys and girls	15-19 years	MUAC	BMI	WHO BMI Z score	overweight	BAZ > +1 SD	27.95	14.5		0.911	0.903	9.3	0.1		112	71	657	11
17	Khiamniungan et.al	2019	India	N/A	cross sectional	960	400	Boys and girls	5-9 years	MUAC	BMI	BMI Z score	overweight	BAZ> +1 SD	16.5	0.5	0.75	0.5	0.653				100	69	131	100
							560	Boys and girls	10-16 years	MUAC	BMI	BMI Z score	overweight	BAZ > +1 SD	21.5	0.2767	0.95	0.948	0.802				147	80	325	8
18	Ozturk et.al	2015	Turkey	N/A	cross sectional	5358	968	Boys	6-10 years	MUAC	WC			≥≥90th percentile	9.5		0.83									
							561	Boys	11-13 years	MUAC	WC			≥≥90th percentile	23.5		0.94									
							301	Boys	31–73 months	MUAC z-score	BMI z-score	IOTF-BMI criteria	Overweight	BMI ≥ 25 kg/m2	NA	9.3%	0.79	66.7	93.0	9.6		0.597				
								Boys	31–73 months	MUAC z-score	BMI z-score	IOTF-BMI criteria	Obesity	BMI ≥ 30 kg/m2	NA	4.7%										
							295	Girls	31–73 months	MUAC z-score	BMI z-score	IOTF-BMI criteria	Overweight	BMI ≥ 25 kg/m2	NA	8.5%	0.94	96.9	89.1	8.9		0.86				
								Girls	31–73 months	MUAC z-score	BMI z-score	IOTF-BMI criteria	Obesity	BMI ≥ 30 kg/m2	NA	6.8%										
19	Ozturk et.al	2015	Turkey	N/A	cross sectional		1092	Boys	14-17 years	MUAC	WC			≥≥90th percentile	25.3		0.9									

Table S4

ID	Primary author	year of publication	Location	Funder	Study design	Total sample size	Sample size / Sub group	Gender	Age (Years)	Index tests (MUAC)	Reference standard	Growth charts	overweight or obesity	Dx criteria according to reference standard	Dx criteria according to MUAC	Prevalence according to reference standard	AUC	Sensitivity	Specificity	LR+	LR-	Youden index	TP	FP	TN	FN
							913	Girls	6-10 years	MUAC	WC			≥≥90th percentile	20.1		0.85									
							577	Girls	11-13 years	MUAC	WC			≥≥90th percentile	23.1		0.91									
							1247	Girls	14-17 years	MUAC	WC			≥≥90th percentile	23.9		0.92									
20	Ramcharitar-Bourne *et al.* [20]	2021	Trinidad and Tobago,	N/A	Cross sectional	596	301	Boys	31–73 months	MUAC z-score	BMI z-score	IOTF-BMI criteria	Overweight	BMI ≥ 25 kg/m2	NA	9.3%	0.79	66.7	93.0	9.6		0.597				
								Boys	31–73 months	MUAC z-score	BMI z-score	IOTF-BMI criteria	Obesity	BMI ≥ 30 kg/m2	NA	4.7%										
							295	Girls	31–73 months	MUAC z-score	BMI z-score	IOTF-BMI criteria	Overweight	BMI ≥ 25 kg/m2	NA	8.5%	0.94	96.9	89.1	8.9		0.86				
								Girls	31–73 months	MUAC z-score	BMI z-score	IOTF-BMI criteria	Obesity	BMI ≥ 30 kg/m2	NA	6.8%										
21	Nitika [21]	2021	India	Nil	Cross sectional	31,471		Boys	10	MUAC	BMI z-score	WHO BMI Z-score	Overweight	BMI≤ +2SD	21.2		0.84	0.745	0.932			0.67				
								Boys	11	MUAC	BMI z-score	WHO BMI Z-score	Overweight	BMI≤ +2SD	21.9		0.88	0.843	0.919			0.76				

Table S4

ID	Primary author	year of publication	Location	Funder	Study design	Total sample size	Sample size / Sub group	Gender	Age (Years)	Index tests (MUAC)	Reference standard	Growth charts	overweight or obesity	Dx criteria according to reference standard	Dx criteria according to MUAC	Prevalence according to reference standard	AUC	Sensitivity	Specificity	LR+	LR-	Youden index	TP	FP	TN	FN
								Boys	12	MUAC	BMI z-score	WHO BMI Z-score	Overweight	BMI≤ +2SD	22.9		0.86	0.798	0.925			0.72				
								Boys	13	MUAC	BMI z-score	WHO BMI Z-score	Overweight	BMI≤ +2SD	24.1		0.86	0.801	0.916			0.72				
								Boys	14	MUAC	BMI z-score	WHO BMI Z-score	Overweight	BMI≤ +2SD	25.3		0.85	0.775	0.926			0.70				
								Boys	15	MUAC	BMI z-score	WHO BMI Z-score	Overweight	BMI≤ +2SD	26.4		0.86	0.788	0.938			0.74				
								Boys	16	MUAC	BMI z-score	WHO BMI Z-score	Overweight	BMI≤ +2SD	27.0		0.88	0.846	0.914			0.77				
								Boys	17	MUAC	BMI z-score	WHO BMI Z-score	Overweight	BMI≤ +2SD	27.7		0.89	0.871	0.906			0.78				
								Boys	18	MUAC	BMI z-score	WHO BMI Z-score	Overweight	BMI≤ +2SD	28.2		0.88	0.861	0.904			0.77				
								Boys	19	MUAC	BMI z-score	WHO BMI Z-score	Overweight	BMI≤ +2SD	29.8		0.94	0.900	0.976			0.88				
								Girls	10	MUAC	BMI z-score	WHO BMI Z-score	Overweight	BMI≤ +2SD	21.2		0.86	0.822	0.898			0.72				
								Girls	11	MUAC	BMI z-score	WHO BMI Z-score	Overweight	BMI≤ +2SD	22.1		0.85	0.803	0.904			0.71				
								Girls	12	MUAC	BMI z-score	WHO BMI Z-score	Overweight	BMI≤ +2SD	23.1		0.84	0.792	0.982			0.69				

Table S4

ID	Primary author	year of publication	Location	Funder	Study design	Total sample size	Sample size / Sub group	Gender	Age (Years)	Index tests (MUAC)	Reference standard	Growth charts	overweight or obesity	Dx criteria according to reference standard	Dx criteria according to MUAC	Prevalence according to reference standard	AUC	Sensitivity	Specificity	LR+	LR-	Youden index	TP	FP	TN	FN
								Girls	13	MUAC	BMI z-score	WHO BMI Z-score	Overweight	BMI≤ +2SD	24.4		0.87	0.819	0.922			0.74				
								Girls	14	MUAC	BMI z-score	WHO BMI Z-score	Overweight	BMI≤ +2SD	24.9		0.89	0.869	0.910			0.78				
								Girls	15	MUAC	BMI z-score	WHO BMI Z-score	Overweight	BMI≤ +2SD	25.2		0.89	0.907	0.883			0.79				
								Girls	16	MUAC	BMI z-score	WHO BMI Z-score	Overweight	BMI≤ +2SD	25.5		0.86	0.841	0.870			0.72				
								Girls	17	MUAC	BMI z-score	WHO BMI Z-score	Overweight	BMI≤ +2SD	26.5		0.89	0.869	0.918			0.79				
								Girls	18	MUAC	BMI z-score	WHO BMI Z-score	Overweight	BMI≤ +2SD	26.5		0.89	0.874	0.912			0.79				
								Girls	19	MUAC	BMI z-score	WHO BMI Z-score	Overweight	BMI≤ +2SD	26.7		0.91	0.889	0.934			0.83				
								Boys	10	MUAC	BMI z-score	WHO BMI Z-score	Obesity	BMI> +2SD	22.4		0.85	0.783	0.926			0.72				
								Boys	11	MUAC	BMI z-score	WHO BMI Z-score	Obesity	BMI> +2SD	23.4		0.86	0.803	0.918			0.72				
								Boys	12	MUAC	BMI z-score	WHO BMI Z-score	Obesity	BMI> +2SD	24.5		0.87	0.800	0.934			0.74				
								Boys	13	MUAC	BMI z-score	WHO BMI Z-score	Obesity	BMI> +2SD	26.4		0.86	0.757	0.962			0.75				

Table S4

ID	Primary author	year of publication	Location	Funder	Study design	Total sample size	Sample size / Sub group	Gender	Age (Years)	Index tests (MUAC)	Reference standard	Growth charts	overweight or obesity	Dx criteria according to reference standard	Dx criteria according to MUAC	Prevalence according to reference standard	AUC	Sensitivity	Specificity	LR+	LR-	Youden index	TP	FP	TN	FN
								Boys	14	MUAC	BMI z-score	WHO BMI Z-score	Obesity	BMI> +2SD	27.2		0.93	0.914	0.950			0.87				
								Boys	15	MUAC	BMI z-score	WHO BMI Z-score	Obesity	BMI> +2SD	27.4		0.94	0941	0.937			0.91				
								Boys	16	MUAC	BMI z-score	WHO BMI Z-score	Obesity	BMI> +2SD	29.7		0.89	0.840	0.928			0.80				
								Boys	17	MUAC	BMI z-score	WHO BMI Z-score	Obesity	BMI> +2SD	29.6		0.92	0.900	0.946			0.85				
								Boys	18	MUAC	BMI z-score	WHO BMI Z-score	Obesity	BMI> +2SD	31.1		0.96	0.944	0.975			0.92				
								Boys	19	MUAC	BMI z-score	WHO BMI Z-score	Obesity	BMI> +2SD	NA		NA	NA	NA			NA				
								Girls	10	MUAC	BMI z-score	WHO BMI Z-score	Obesity	BMI> +2SD	23.9		0.83	0.688	0.971			0.69				
								Girls	11	MUAC	BMI z-score	WHO BMI Z-score	Obesity	BMI> +2SD	24.2		0.95	0.950	0.948			0.90				
								Girls	12	MUAC	BMI z-score	WHO BMI Z-score	Obesity	BMI> +2SD	25.7		0.89	0.821	0.956			0.78				
								Girls	13	MUAC	BMI z-score	WHO BMI Z-score	Obesity	BMI> +2SD	26.6		0.89	0.12	0.958			0.77				
								Girls	14	MUAC	BMI z-score	WHO BMI Z-score	Obesity	BMI> +2SD	27.6		0.91	0.844	0.973			0.82				

Table S4

ID	Primary author	year of publication	Location	Funder	Study design	Total sample size	Sample size / Sub group	Gender	Age (Years)	Index tests (MUAC)	Reference standard	Growth charts	overweight or obesity	Dx criteria according to reference standard	Dx criteria according to MUAC	Prevalence according to reference standard	AUC	Sensitivity	Specificity	LR+	LR-	Youden index	TP	FP	TN	FN
								Girls	15	MUAC	BMI z-score	WHO BMI Z-score	Obesity	BMI> +2SD	28.0		0.89	0.809	0.964			0.77				
								Girls	16	MUAC	BMI z-score	WHO BMI Z-score	Obesity	BMI> +2SD	27.3		0.89	0.833	0.942			0.82				
								Girls	17	MUAC	BMI z-score	WHO BMI Z-score	Obesity	BMI> +2SD	29.1		0.93	0.893	0.976			0.87				
								Girls	18	MUAC	BMI z-score	WHO BMI Z-score	Obesity	BMI> +2SD	29.9		0.88	0.778	0.979			0.81				
								Girls	19	MUAC	BMI z-score	WHO BMI Z-score	Obesity	BMI> +2SD	NA		NA	NA	NA			NA				

Table S5

Study	RISK OF BIAS				APPLICABILITY CONCERNS		
	PATIENT SELECTION	INDEX TEST	REFERENCE STANDARD	FLOW AND TIMING	PATIENT SELECTION	INDEX TEST	REFERENCE STANDARD
Lu *et al.* [(1)]	Low	Low	High	Low	Low	Low	Low
Rerksuppaphol & Rerksuppaphol [(2)]	Low	Low	High	Low	Low	Low	Low
Asif *et al.* [(3)]	Low	Low	High	Low	Low	Low	Low
Craig *et al.* [(4)]	Low	Low	High	Low	Low	Low	Low
Chaput *et al.* [(5)]	Low	Low	High	Low	Low	Low	Low
Jaiswal *et al.* [(6)]	Low	Low	High	Low	Low	Low	Low
Talma *et al.* [(7)]	Low	Low	High	Low	Low	Low	Low
Mazıcıoğlu *et al.* [(8)]	Low	Low	High	Low	Low	Low	Low
Ayu *et al.* [(9)]	Low	Low	High	Low	Low	Low	Low
Shinsugi *et al.* [(10)]	Low	Low	High	Low	Low	Low	Low
Dumith *et al.* [(11)]	Low	Low	High	Low	Low	Low	Low
Otitoola *et al.* [(12)]	Low	Low	High	Low	Low	Low	Low
Orimadegun [(13)]	Low	Low	High	Low	Low	Low	Low
Asif *et al.* [(14)]	Low	Low	High	Low	Low	Low	Low
de Almeida *et al.* [(15)]	Low	Low	High	Low	Low	High	High
Oriaifo *et al.* [(16)]	Low	Low	High	Low	Low	Low	Low
Sisay *et al.* [(17)]	Low	Low	High	Low	Low	Low	Low
Khiamniungan & Mondal [(18)]	Low	Low	High	Low	Low	Low	Low
Ozturk *et al.* [(19)]	Low	Low	High	Low	Low	Low	Low
Ramcharitar-Bourne *et al.* [(20)]	Low	Low	High	Low	Low	Low	Low
Nitika [(21)]	Low	Low	High	Low	Low	Low	Low

1. Lu Q, Wang R, Lou DH *et al.* (2014) Mid-upper-arm circumference and arm-to-height ratio in evaluation of overweight and obesity in Han children. *Pediatr Neonatol* 55, 14-19.
2. Rerksuppaphol S & Rerksuppaphol L (2017) Mid-Upper-Arm Circumference and Arm-to-Height Ratio to Identify Obesity in School-Age Children. *Clin Med Res* 15, 53-58.
3. Asif M, Aslam M Altaf S (2018) Mid-upper-arm circumference as a screening measure for identifying children with elevated body mass index: a study for Pakistan. *Korean J Pediatr* 61, 6-11.
4. Craig E, Bland R, Ndirangu J *et al.* (2014) Use of mid-upper arm circumference for determining overweight and overfatness in children and adolescents. *Arch Dis Child* 99, 763-766.
5. Chaput JP, Katzmarzyk PT, Barnes JD *et al.* (2017) Mid-upper arm circumference as a screening tool for identifying children with obesity: a 12-country study. *Pediatr Obes* 12, 439-445.
6. Jaiswal M, Bansal R Agarwal A (2017) Role of Mid-Upper Arm Circumference for Determining Overweight and Obesity in Children and Adolescents. *J Clin Diagn Res* 11, Sc05-sc08.
7. Talma H, van Dommelen P, Schweizer JJ *et al.* (2019) Is mid-upper arm circumference in Dutch children useful in identifying obesity? *Arch Dis Child* 104, 159-165.
8. Mazıcıoğlu MM, Hatipoğlu N, Oztürk A *et al.* (2010) Waist circumference and mid-upper arm circumference in evaluation of obesity in children aged between 6 and 17 years. *J Clin Res Pediatr Endocrinol* 2, 144-150.
9. Ayu DR, Aditiawati A, Anzar J *et al.* (2017) Upper arm circumference measurement for detecting overweight and obesity in children aged 6-7 years. *Paediatr Indones* 57, 23-29.
10. Shinsugi C, Gunasekara D Takimoto H (2020) Use of Mid-Upper Arm Circumference (MUAC) to Predict Malnutrition among Sri Lankan Schoolchildren. *Nutrients* 12.
11. Dumith SC, Muraro MFR, Monteiro AR *et al.* (2018) Diagnostic properties and cutoff points for overweight prediction through anthropometric indicators in adolescents from Caracol, Piauí, Brazil, 2011. *Epidemiol Serv Saude* 27, e201715013.
12. Otitoola O, Oldewage-Theron W Egal A (2020) Prevalence of overweight and obesity among selected schoolchildren and adolescents in Cofimvaba, South Africa. *South Afr J Clin Nutr*, 1-6.
13. Orimadegun A (2019) Accuracy of mid upper arm circumference in detection of obesity among school children in Yenagoa City, South-south region of Nigeria. *Nigerian journal of paediatrics* 46, 48-54.
14. Asif M, Muhammad A Altaf S (2018) Use of mid-upper arm circumference in evaluation of overweight and obesity in the Pakistani children and adolescent, aged 12-18 years. *Pakistan Paediatric Journal* 42, 43-48.
15. de Almeida CA, Del Ciampo LA, Ricco RG *et al.* (2003) [Assessment of mid-upper arm circumference as a method for obesity screening in preschool children]. *J Pediatr (Rio J)* 79, 455-460.

16. Oriaifo S, Abiodun P, Atimati A *et al.* (2019) Determination of overnutrition using mid-upper arm circumference in comparison with bioelectrical impedance analysis in children and adolescents in Benin, Nigeria. *Journal of Health Research* ahead-of-print.
17. Sisay BG, Haile D, Hassen HY *et al.* (2020) Performance of mid-upper arm circumference as a screening tool for identifying adolescents with overweight and obesity. *PLoS One* 15, e0235063.
18. Khiamniungan KS & Mondal PR (2019) Mid-Upper Arm Circumference: An Alternative to BMI for Screening Overweight and Obesity Among the Khiamniungan Tribal Children and Adolescents of Nagaland, Northeast India. *Online Journal of Health & Allied Sciences* 18, 1-4.
19. Ozturk A, Cicek B, Mazicioglu MM *et al.* (2015) Determining abdominal obesity cut-offs and relevant risk factors for anthropometric indices in Turkish children and adolescents. *J Pediatr Endocrinol Metab* 28, 525-532.
20. Ramcharitar-Bourne A, Nichols SD Badrie N (2021) Predictive utility of anthropometric based cut-offs in assessing excess adiposity among preschool children in a multiethnic population. *West Indian Medical Journal* 69, 114-120.
21. Nitika N (2021) Discriminatory ability of mid-upper arm circumference in identifying overweight and obese adolescents: Findings from the comprehensive national nutrition survey, India. *Indian Journal of Public Health* 65, 269-274.

Table S6

Question: Should Mid-Upper Arm Circumference (MUAC) be used to diagnose overweight and obesity in children and adolescents?

Sensitivity	0.85 (95% CI: 0.77 to 0.91)
Specificity	0.86 (95% CI: 0.81 to 0.98)

Prevalence of overweight and obesity	13.14%		

Outcome	№ of studies (№ of patients)	Study design	Factors that may decrease certainty of evidence					Effect per 1,000 patients tested	Test accuracy CoE
			Risk of bias	Indirectness	Inconsistency	Imprecision	Publication bias	pre-test probability of13.14%	
**True positives** (patients with Overweight and obesity)	9 studies 2877 patients	cross-sectional (cohort type accuracy study)	serious[a]	serious[b]	not serious	not serious	strong association	112 (102 to 119)	⨁⨁⨁◯ Moderate
**False negatives** (patients incorrectly classified as not having Overweight and obesity)								19 (12 to 29)	
**True negatives** (patients without Overweight and obesity)	9 studies 18592 patients	cross-sectional (cohort type accuracy study)	serious[a]	serious[b]	not serious	not serious	strong association	744 (704 to 853)	⨁⨁⨁◯ Moderate
**False positives** (patients incorrectly classified as having Overweight and obesity)								125 (16 to 165)	

Explanations

a. All the included studies compare the performance of MUAC with BMI in identifying children and adolescents with overweight and obesity. BMI is not a golden standard to measure excess adiposity. Even though BMI is highly correlated with percent body fat it does not differentiate between lean mass and fat mass and fail to identify a significant number of children with overweight and obesity.

b. All the included studies compare the performance of MUAC with BMI in identifying children and adolescents with overweight and obesity. BMI is not a direct measure of excess adiposity; it indirectly measures excess adiposity. Hence, fail to identify a significant number of children with overweight and obesity.
